# Species Accumulation Stabilizes the Synchronous Responses to the Environment of Vertebrate Communities Worldwide

**DOI:** 10.1002/ece3.73723

**Published:** 2026-06-21

**Authors:** Sergio Picó, Pablo Almaraz, Oscar Godoy

**Affiliations:** ^1^ Departamento de Biología Instituto Universitario de Investigación Marina (INMAR), Universidad de Cádiz Puerto Real Spain; ^2^ Estación Biológica de Doñana (EBD‐CSIC) Sevilla Spain; ^3^ Department of Ecology and Coastal Management Instituto de Ciencias Marinas de Andalucía (ICMAN‐CSIC), Campus Universitario de Puerto Real Puerto Real Spain

**Keywords:** compensatory dynamics, stability, synchrony, terrestrial vertebrates

## Abstract

Multiple drivers contribute to community stability measured as the temporal invariability of total abundance, density, or biomass. However, the direct and indirect effects of these drivers, such as number of species, synchrony between species, species' life strategies, and environmental conditions in communities of terrestrial vertebrates remain unclear. To address this gap, we compiled a worldwide dataset of time series corresponding to 124 natural communities of terrestrial vertebrates including mammals, birds, and reptiles. We found that communities tend to be more synchronous than expected under the theoretical scenario of weak covariance between species because, at the interannual level, shared responses to the environment predominate over compensatory dynamics. Furthermore, piecewise structural equation models reveal that the number of species had a consistent positive effect on stability (either directly or indirectly via synchrony), but the sign and magnitude of the paths involving synchrony changed depending on the metric used and whether detrending was applied or not. Interestingly, we did not find the expected decreasing latitudinal gradient in community stability towards the poles. Our results demonstrate that the synchrony metrics used are not interchangeable and thus, whenever we use multivariate methods to understand the relation between richness, synchrony, and stability, our results may depend on how we define synchrony and how we measure it. Put together, our results also reinforce the idea of the predominance of environmental forcing over compensatory dynamics and the key role of the accumulation of species (statistical averaging) for the temporal stability of ecological communities.

## Graphical abstract

How species number, synchrony, species life strategies, and environmental conditions interact and contribute to community stability is still unclear. We compiled a worldwide dataset of time series from 124 natural communities of terrestrial vertebrates and found that, while they tend to be mostly synchronous, species accumulation helped to maintain community stability. Although the exact balance of effects depended on the metric used and if detrending of the time series was applied.

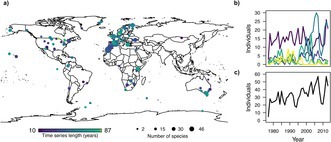

## 1 Introduction

Stability has been a central topic in community ecology since its inception (Holling 1973; MacArthur 1955; Pimm 1984) and this extensive history has given rise to numerous measures and definitions of stability (Domínguez‐García et al. 2019; Donohue et al. 2016). One of the simplest forms of community stability is invariability, the inverse of its temporal variability, calculated with the inverse of the coefficient of variation of the total abundance in the community (Tilman 1996). Theory and empirical evidence have shown that the temporal stability of ecological communities is crucial for providing multiple services critical to human well‐being, such as food production (Balvanera et al. 2006). Given that human activities are causing unprecedented changes in natural communities and ecosystems that affect their ability to resist perturbations and recover after them (Hautier et al. 2015; Vitousek et al. 1997), understanding the drivers of community stability is more important than ever.

A community can achieve stability in the sense of invariability, that is, a low coefficient of variation of total abundance, by the action of three different mechanisms: (1) By the presence of stable dominant species. This so‐called dominance effect determines the stability of the community when a species or a few species dominate the total abundance in a community (Hillebrand et al. 2008). (2) By accumulation of species, or statistical averaging. That is, adding more species to the sum will stabilize total abundance, provided that their dynamics are not perfectly correlated and regardless of the covariance between species (Doak et al. 1998). (3) By anti‐synchronous fluctuations, or compensatory dynamics. In this phenomenon, the differences in the direction, magnitude, and timing of species responses to changes in the environment—often mediated by species interactions—cause negative covariances between the fluctuations of the populations of different species, which tend to cancel out, thus stabilizing total abundance (Gonzalez and Loreau 2009; Loreau and de Mazancourt 2013).

The interplay between dominance effects, statistical averaging, and compensatory dynamics has been pointed out as the key mechanism generating the observed biodiversity insurance or portfolio effect (Thibaut and Connolly 2013; Yachi and Loreau 1999). This is the case by which a high number of species would stabilize the aggregated properties of the community by guaranteeing a good performance—i.e., high population densities—even if some species fail to do so due to changes in environmental conditions. Thus, there have been many attempts at measuring these mechanisms, frequently grouping them under the ambiguous term “synchrony” and, as a consequence, creating a myriad of different “synchrony” metrics that measure different facets of the same phenomenon (Bjørkås et al. 2025; Gross et al. 2014; Loreau and de Mazancourt 2008; Segrestin et al. 2024; Shoemaker et al. 2022).

In particular, anti‐synchronous fluctuations—generally understood as the case where two or more taxa or populations show negatively correlated dynamics—have received much attention in the last two decades but, while there are many examples of how they can contribute to the stability of aggregate properties of theoretical communities (Gonzalez and Loreau 2009), their importance in the real world is much less understood. Empirical support for anti‐synchronous fluctuations in natural communities has been scarce (Ernest et al. 2008; Vasseur and Gaedke 2007), compared to synchronous dynamics—often measured as positive correlations or covariances—that seem to be the norm (Houlahan et al. 2007; Mutshinda et al. 2009; Vasseur et al. 2014). As a result, the degree of prevalence of a positive relationship between richness and temporal stability, and what drivers modulate this relation, are still controversial. To shed some light on this area, we first reviewed which synchrony metrics were the most used in the last 5 years in some of the main ecology journals (Figure S2). With the information provided by the review, we decided to use the two most popular ones, the phi (ϕ) index (Loreau and de Mazancourt 2008) and the eta (η) index (Gross et al. 2014) as representative examples of simple “synchrony metrics”. Although both are widely used to measure synchrony, they do so from alternative approaches. While ϕ measures how much of the variability at the species level is conserved at the community level (Loreau and de Mazancourt 2008)—a “stabilization” or “compensation” effect—, η measures mean correlation between species (Gross et al. 2014). Therefore, because these two widely used metrics differ in how species richness affects measures of synchrony, they give us different but complementary information about contrasting aspects of community synchrony.

The main use of synchrony metrics has been to detect statistical averaging and/or anti‐synchronous fluctuations in the field. Interestingly, natural communities tend to be mostly synchronous (Houlahan et al. 2007; Mutshinda et al. 2009; Vasseur et al. 2014). Why is this the case when anti‐synchronous fluctuations appear frequently in theoretical models and controlled experiments? Environmental fluctuations—such as variability in temperature and precipitation—can also impact species and community dynamics through their effects on species vital rates and species interactions (Mutshinda et al. 2009). This effect of “environmental forcing” could create synchronous fluctuations that result in variability in total community size, even when statistical averaging and anti‐synchronous fluctuations occur (Ranta et al. 2008). On the other hand, some of the theoretical models that generate anti‐synchronous fluctuations rely on a zero‐sum system (Loreau and de Mazancourt 2008), like a fixed total community size or a fixed resource availability, while that is not the case in most natural systems. Another obscuring factor is timescales. Time‐series data are usually collected once every year and just for a few years in most animal and plant studies, but anti‐synchronous fluctuations could happen at a shorter or longer timescale (Luo et al. 2025; Shoemaker et al. 2022). For example, given a certain study length and sampling periodicity, we could expect more temporal variability in a community of rodents in the Arctic—that experiences strong seasonal fluctuations—than in a community of ungulates in an African savanna—that may fluctuate with a multi‐year period—due to their different sensitivity to variability in vital rates (Morris et al. 2008). The taxonomic level used as the unit of analysis could also create another problem: anti‐synchronous fluctuations could happen between groups of ecologically similar species or guilds (Barraquand et al. 2022), and not between species.

The mechanisms of synchrony and stability in natural communities and how these are modified by temperature and precipitation have been extensively analyzed in plants (Gu et al. 2023; Hallett et al. 2014; Kigel et al. 2021; Lisner et al. 2024; Ma et al. 2017; Tilman 1996; Tredennick et al. 2017; Valencia, de Bello, Galland, et al. 2020; Wang et al. 2020; Zhao et al. 2022), with some examples in other organisms like invertebrates (Larsen et al. 2024; van Klink et al. 2019, 2022) or plankton (Vasseur and Gaedke 2007; Vasseur et al. 2014). However, the research on other groups, such as the case of terrestrial vertebrates, is scarcer. This scarcity is due to data limitations because studies are usually conducted at the local scale (Barraquand et al. 2022; Ernest et al. 2008; Li et al. 2021; White et al. 2023), and terrestrial vertebrates are often a small proportion of the data in studies with a wider taxonomic range (Houlahan et al. 2007; Mutshinda et al. 2009).

**FIGURE 1:**
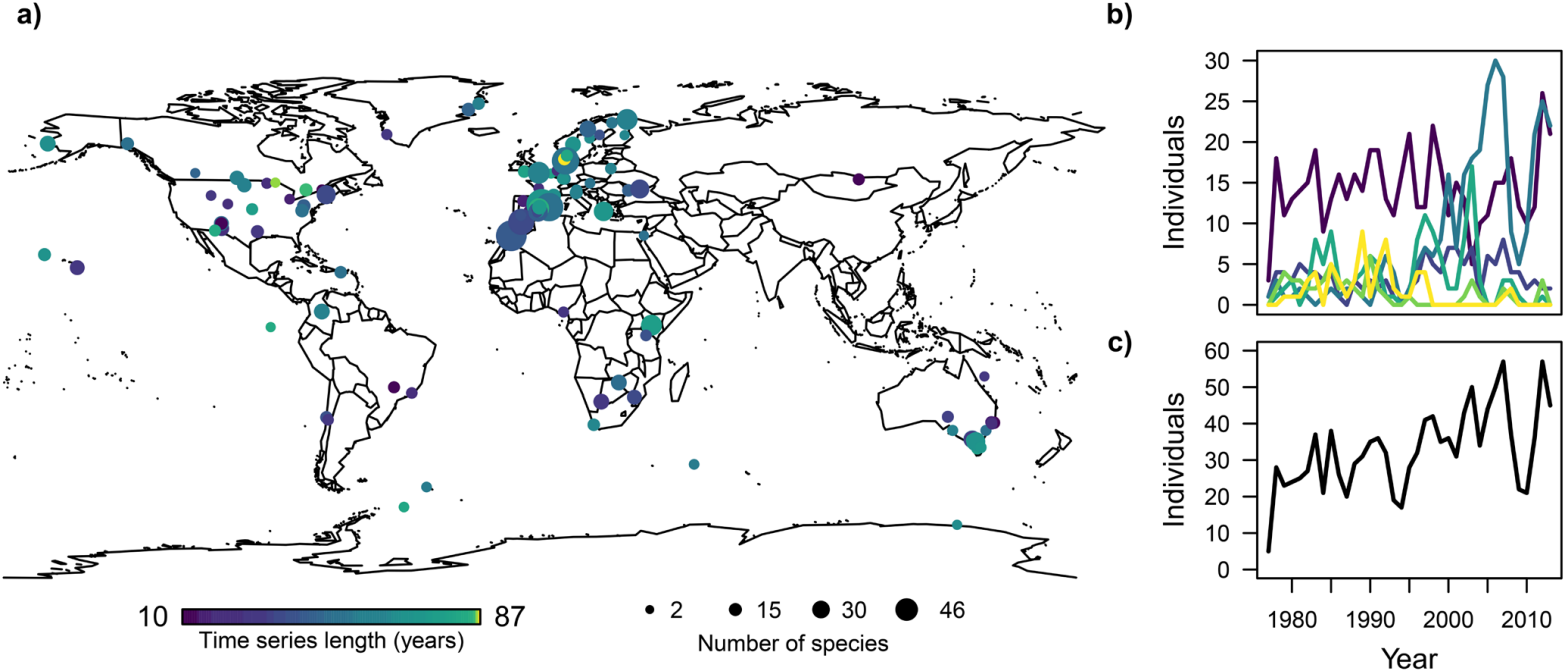
Summary of the dataset. (a) Global map showing the distribution of the communities considered. The color of points represents the length of the time series, and their size represents the number of species included. (b) Time series for the species included in a community of rodents monitored at Portal, Arizona (USA). (c) Time series for total abundance in the same community.

Here, we aim to shed light on the main mechanisms driving the temporal stability of natural communities of terrestrial vertebrates and the synchrony between their species. To do this, we compiled a global database of 124 natural communities focusing on terrestrial vertebrates (birds, mammals, and reptiles) (Figure 1). Coupled with this database, we collected climatic data representing the mean environmental context at the location of each community (Figure S3), as well as longevity data, both mean and coefficient of variation as proxies of species' life strategies. Our main question to evaluate by means of piecewise structural equation models (SEMs) is the following: which are the direct and indirect paths by which number of species, species synchrony, species lifespan, and environmental conditions determine the temporal stability of these communities? According to prior work, our four main hypotheses were (1) Higher stability and lower synchrony will occur in communities with more species, because of the portfolio effect (Xu et al. 2021); (2) We expect a negative relation between latitude and stability, due to increasing population fluctuations caused by the variability of environmental conditions (Dallas and Kramer 2021); (3) Higher stability will arise in communities with longer‐lived species because their populations are less sensitive to environmental variation compared to shorter‐lived species (Morris et al. 2008) and in those communities with more diversity of life strategies (represented by interspecific variability in lifespan) because it promotes anti‐synchrony (White et al. 2023).

## 2 Methods

### 2.1 Database Compilation: Time Series, Environmental Data, and Species' Lifespan

We compiled a database of time series of abundance, densities, or counts from local natural communities of terrestrial vertebrates, defined as at least two species living in the same defined area and simultaneously monitored using measures of abundance or density. We searched existing databases such as BioTIME (Dornelas et al. 2018) and The Living Planet Index (LPI 2022; Loh et al. 2005), the scientific literature, and the gray literature. We aimed for studies that followed one or more of these communities annually for at least 10 consecutive years using consistent methods. It is important to note that the number of species included in these studies does not necessarily reflect the real richness of the group studied; therefore, we refer to it as the number of species rather than richness to avoid confusion. We chose this threshold because it has been shown that 10 years is often enough to detect population trends or synchrony patterns (Luo et al. 2025; White 2019), and it results in a sufficient amount of datasets available. Of the more than 300 studies checked, we finally obtained 124 communities with a minimum of 2 and a maximum of 46 species monitored during a mean time of 22 years (range 10–87 years), covering a wide geographical and climatic range (Figure 1, Figure S3). We did not include datasets in which counts or densities were not the raw information, excluding other measures such as unit‐less indices. Data were extracted from the actual sources and checked to ensure the data fulfilled our requirements. Raw data were recovered from plots when needed using WebPlotDigitizer 4.5 (Rohatgi 2022). A detailed list of the selected communities and their sources is included in the shared data folder (https://doi.org/10.6084/m9.figshare.30455942), and a summary of the number of species, study duration, and taxa included in the communities studied is shown in Figure S1.

We selected lifespan as a key trait that explains the life history of species, their response to environmental variability, and potentially the variability of their populations (Morris et al. 2008). We obtained lifespan data from a database of amniote life‐history traits (Myhrvold et al. 2015). Lifespan data were available for 103 communities with at least 75% coverage of populations, covering 97.08% of all populations in these communities. We then calculated the mean lifespan weighted by species relative abundance for every community and the coefficient of variation of the lifespan values for species in every community using standard deviation and mean, also weighted by relative abundance.

We obtained climatic data for each community's location from the Chelsa Climate database (Brun et al. 2022a, 2022b) using the geographic coordinates (in degrees) of the study or its approximated centroid when the exact coordinates were not defined. We selected mean annual temperature (bio1, °C) and mean annual precipitation (bio12, kg/m^2^, sum of monthly precipitation totals) as the variables that describe the mean environmental conditions, or environmental context, that populations experience in each study area. We selected them because mean temperature and annual precipitation are key variables that define a particular climate or, as in Whittaker's classification, a biome (Figure S3).

### 2.2 Temporal Stability Analysis: Synchrony Metrics and Piecewise Structural Equation Modeling

For each community, we calculated temporal stability as the inverse of its coefficient of variation $1/\mathrm{CV}\left(A_{\mathrm{tot}}\right)=\text{mean}\left(A_{\mathrm{tot}}\right)/\mathrm{sd}\left(A_{\mathrm{tot}}\right)$ where $\text{mean}\left(A_{\mathrm{tot}}\right)$ corresponds to the mean of total abundance in the community, and $\mathrm{sd}\left(A_{\mathrm{tot}}\right)$ corresponds to the standard deviation of the same variable. To account for the possible limitations of the coefficient of variation, we also calculated the temporal stability of total abundance using the inverse of proportional variation (Heath 2006)

(1) $$\mathrm{PV}=\frac{1}{C}\sum_{\text{comb}}D\left(z_{i},z_{j}\right)$$

where *C* is the number of unique pairwise combinations of values in the time series and $D\left(z_{i},z_{j}\right)$ the relative difference between a pair of values. The relative difference $D\left(z\right)$ between two values $z_{i}$, $z_{j}$, is defined as

(2) $$\left\{\begin{array}{ll} 0, & \text{if}\;z_{i}=z_{j}\\ \frac{\mathrm{abs}\left(z_{i}-z_{j}\right)}{\max\left(z_{i},z_{j}\right)}, & \text{if}\;z_{i}\neq z_{j} \end{array}\right.$$

PV can, in theory, help to control for the differences in length of the time series and for non‐Gaussian data. Both stability measures were highly correlated (Figure S4, Spearman's ρ = 0.98), so we only show results using 1/cv because it is easier to interpret.

To assess the degree of synchrony between species, we calculated for each community two of the synchrony metrics most widely used, ϕ and η (Figure S2). We determined the proportion of use of these metrics reviewing all measures of synchrony used in the last 5 years (2020–2024) in the journals *Ecography*, *Ecology*, *Ecology Letters*, *Global Ecology and Biogeography*, *Journal of Animal Ecology*, *Journal of Ecology*, *Nature Ecology and Evolution*, and *The American Naturalist* by filtering articles that included the term synchron‐ or asynchron‐ in the title or the abstract using Web of Science and then choosing the relevant ones. ϕ is defined as the ratio between the variance of the total sum of abundance or density in the communities and the square of the sum of standard deviations for each species (Loreau and de Mazancourt 2008)

(3) $$\phi =\sigma_{x_{T}}^{2}/{\left(\sum_{i}\sigma_{x_{i}}\right)}^{2}$$

where $\sigma_{x_{T}}^{2}$ is the variance of total abundance in the community and $\sigma_{x_{i}}$ the standard deviation of species *i*. This index can also be interpreted as the square of the ratio between the coefficient of variation of total abundance or density and the weighted coefficients of variation of all species (Thibaut and Connolly 2013). Its range goes from 0 (*perfect anti‐synchrony*) to 1 (*perfect synchrony*), and it suffers from the limitation that, with independent species fluctuations, its value will be determined by the number of species. Meanwhile, η is the average across species of the correlation between the abundance or density of each species and the sum of the abundance or density of all other species

(4) $$\eta =\frac{1}{n}\sum_{i}\left(\text{corr}\left(Y_{i},\sum_{j\neq i}Y_{j}\right)w_{i}\right)$$

where $Y_{i}$ is the abundance of species *i*, with relative abundance $w_{i}$ in a community with *n* species. It ranges between −1 (perfect anti‐synchrony) and + 1 (perfect synchrony) and is expected to be centered at 0 with independent species fluctuations (Gross et al. 2014). We calculated η following Blüthgen et al. (2016) as the weighted average correlation between the abundance of every species and the sum of the abundance of all the other species in the community.

Opposite directional trends or opposite long‐term fluctuations for some species in a community can affect estimations of synchrony irrespective of year‐to‐year fluctuations, as has been shown in plant communities (Luo et al. 2025; Valencia, de Bello, Lepš, et al. 2020). To control for this, we applied two different approaches proposed by Lepš et al. (2019). First, we used a method of decomposition of trend and detrended synchrony. The first step is to calculate a linear regression of the observed values in the time series versus time. Then, the total sum of squares can be decomposed into the regression and residual sum of squares. The synchrony measure obtained (S) is defined as the sum of all covariances divided by the expected variance.

(5) $$\text{Stotal}=\frac{2\sum_{i,j>i}^{\mathrm{nsp}}\text{covar}\left(x_{\mathrm{ij}}\right)}{\sum_{i=1}^{\mathrm{nsp}}\mathrm{var}\left(x_{i}\right)}$$

Positive values indicate synchrony (predominance of positive covariances), and negative values indicate anti‐synchrony (prevalence of negative covariances). Using the covariance of the values predicted by the linear regression, we obtain the part of synchrony generated by the trend (Strend), and using the covariance of the residuals, we capture the synchrony not due to trends (Sdetrended). Thus, total synchrony can be decomposed as follows:

(6) Stotal=Strend+Sdetrended

This first method cannot be applied to ϕ or η, thus we also filtered out the effects of trends and long‐term dynamics in these metrics using the three‐year window method proposed in Lepš et al. (2019), that uses the three‐term local variance (T3) defined as

(7) $$T3=\frac{\sum_{i}^{n-2}{\left(x_{i}-2x_{i+1}+x_{i+2}\right)}^{2}}{6\left(n-2\right)}$$

where *n* is the number of years in the time series, *i* is the index of the year and *x* 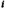 the abundance of a species in year *i*. T3 can then be applied to ϕ by modifying the way variance and covariance are computed and to η by replacing the variances (Lepš et al. 2019).

We generated a set of synthetic community time series to serve as a benchmark for the observed synchrony values under three extreme scenarios: (1) Weak covariance, (2) High positive covariance, and (3) High negative covariance. These simulated communities allow us to establish the theoretical range of synchrony values attainable for each number of species and to asses where the observed values fall within this range. Although some of these limits are unlikely to be reached in nature, they provide useful reference points. For each simulated community, we generated a random correlation matrix with diagonal elements set to one and off‐diagonal elements drawn uniformly from $\left[-0.1,0.1\right]$ and $\left[0.9,1\right]$ for the weak and positive covariance scenarios, respectively. For the negative covariance scenario, the target correlation was set to $-\alpha /\left(S-1\right)$ with α=0.9, which represents the most negative feasible correlation for S species while satisfying the positive‐definiteness constraint of the correlation matrix (a valid SxS correlation matrix with uniform off‐diagonal elements ρ requires $\rho >-1/\left(S-1\right)$). We ensured positive definiteness via eigenvalue clipping and converted each correlation matrix into a variance–covariance matrix Σ by applying random standard deviations to each species. Species abundances were drawn at each time step from a multivariate normal distribution $n_{t}∼N\left(\bar{n}\cdot 1,\Sigma \right)$ where $\bar{n}=10$ is the mean abundance for all species. For each scenario, we simulated 200 communities for each of 13 levels between 2 and 50 species, with 200 time steps per community. Lastly, we calculated the mean and 95% confidence interval for the ϕ and η metrics at each level of number of species.

We used piecewise structural equation modeling (SEM) (Shipley 2000) in combination with spatial simultaneous auto‐regressive lag models to evaluate the multiple direct and indirect effects of number of species, synchrony, lifespan, and environmental conditions on community stability. SEM has been widely used in the past by studies that deal with the relations between richness, synchrony, and community stability (Hallett et al. 2014; Valencia, de Bello, Galland, et al. 2020). We additionally coupled SEM with auto‐regressive lag models to account for the spatio‐temporal nature of our worldwide data. We proposed an a priori model based on previous research (Dallas and Kramer 2021; Morris et al. 2008; White et al. 2023; Xu et al. 2021), in which number of species, the coefficient of variation of lifespan, annual mean temperature, and annual mean precipitation affect synchrony, and synchrony, mean lifespan, mean annual precipitation, and annual mean temperature affect stability directly. Latitude measured as the absolute value of latitude in degrees affects annual mean temperature and annual mean precipitation (Figure S9). The spatial simultaneous auto‐regressive lag models used have the following structure:

(8) y=ρWy+Xβ+ϵ

where *y* represents the stability of communities, ρ the effect of spatial proximity, *W* the distances matrix, *X* the matrix of explanatory variables, β their effects on *y*, and ϵ the stochastic component (the impact of a set of independent and identically distributed random variables with 0 mean and variance $\sigma^{2}$). In this analysis, we included all communities for which climatic data and lifespan data with enough coverage were available (103 communities in total). All variables were log‐transformed and scaled prior to analyses. We evaluated our apriori model 6 times, using ϕ, η, detrended ϕ, detrended η, Stotal and Sdetrended. Then, we added the paths suggested by the directed separation (d‐separation) tests. Briefly, this procedure tests the assumption that there are no missing relationships among unconnected variables (Shipley 2000). All analyses were performed using R 4.1.2 (R Core Team 2022) and packages tempo (Lepš et al. 2019), spdep (Pebesma and Bivand 2023), spatialreg (Pebesma and Bivand 2023), and piecewiseSEM (Lefcheck 2016).

## 3 Results

**FIGURE 2:**
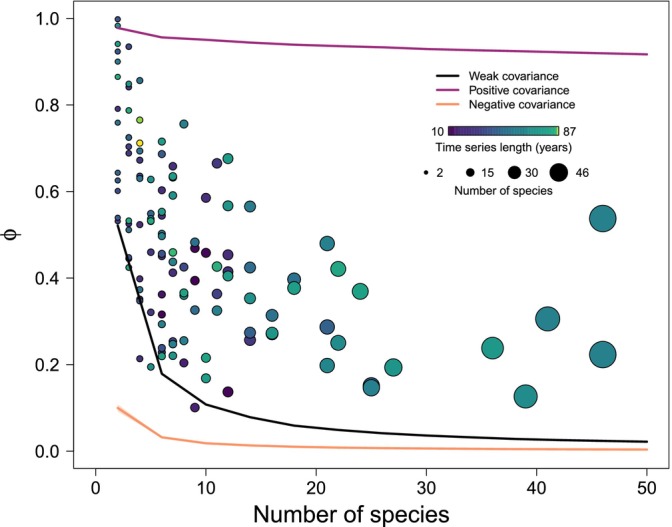
Values of non‐detrended ϕ observed and theoretical expectations depending on the number of species in the community. Lines represent the mean value expected for each theoretical case along the number of species gradient, and shaded areas represent the 95% confidence interval for those means. Each line represents one of three possible extreme cases: Weak covariance, positive covariance, and negative covariance. Each point represents the value for an observed community. Point size represents the number of species considered, and color represents the length of the study in years.

We found that the analyzed communities were mostly synchronous, that is, the empirical values of both ϕ and η were above the expected values for the case of weak covariance between species (Figures 2, S6, 3, S7), and the values of S were also mostly above 0 (Figure S8). Similarly, both metrics indicated that the range of possible synchrony values is maximum at the lowest values of number of species, and it shrinks with species accumulation, leading to values close to 0 in both metrics. For ϕ, this pattern shows how, as number of species increases, stabilization by statistical averaging becomes increasingly likely. Meanwhile, for η this pattern shows how, as number of species increases, strong mean correlation—either positive or negative—becomes unlikely. When we classified communities depending on their combination of ϕ and η values (Figure S5), we observed that most of them are located in the upper left (A) quadrant (46.0% of the total), suggesting that in most cases small differences in population fluctuations stabilize the total abundance even with average positive correlation, especially in communities with many species. Lower stabilization and positive correlation were also common (quadrant B, 37.9% of the total). Negative correlation and high compensation—the classic example of compensatory dynamics illustrated in the C quadrant—was rare, while the case of negative correlation and low compensation (quadrant D) was represented by a few communities that separate themselves from the rest of the cloud of points and have in common that they all contain only a few species and one of them dominates total abundance.

**FIGURE 3:**
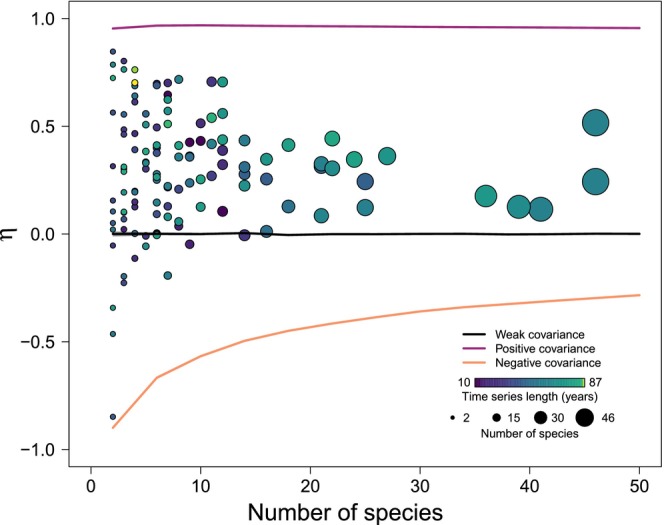
Values of non‐detrended η observed and theoretical expectations depending on the number of species in the community. Lines represent the mean value expected for each theoretical case along the number of species gradient, and shaded areas represent the 95% confidence interval for those means. Each line represents one of three possible extreme cases: Weak covariance, positive covariance, and negative covariance. Each point represents the value for an observed community. Point size represents the number of species considered, and color represents the length of the study in years.

**FIGURE 4:**
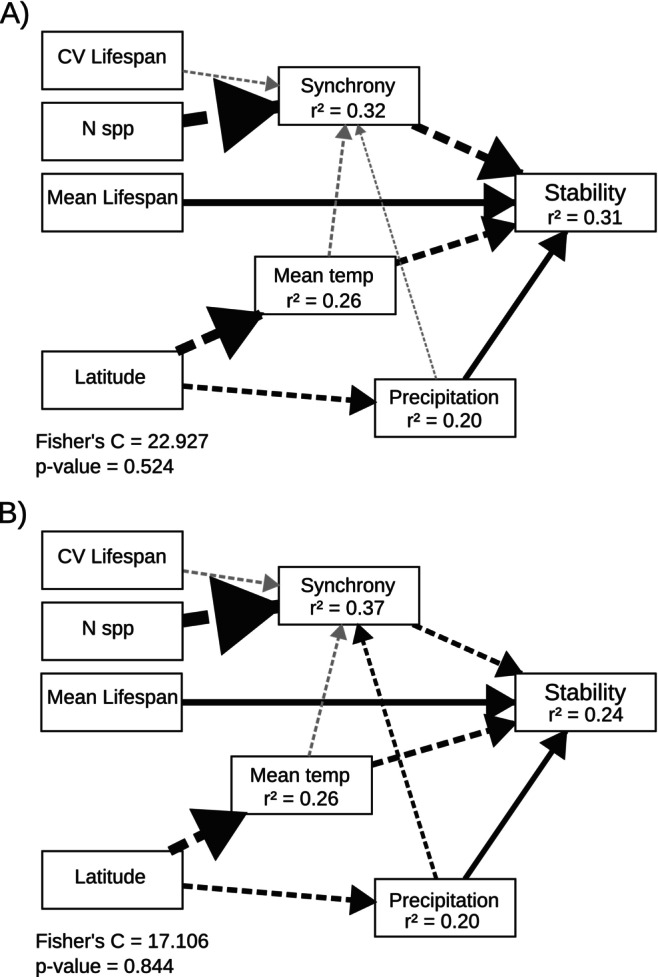
Piecewise structural equations models using ϕ as synchrony metric. (A) Model using non‐detrended ϕ. (B) Model using detrended ϕ. Solid lines represent positive effects while dashed lines represent negative effects. Black lines represent significant effects according to the tests of d‐separation, gray lines nonsignificant effects. The width of each arrow is proportional to the corresponding standardized path coefficient.

In the main results section, we show only the piecewise structural equation models using ϕ for clarity. The model using non‐detrended ϕ (Figure 4A) explained 31% of the variability in stability. The number of species within the community had a positive effect on stability (0.17, Table S1) through two negative paths from species number to synchrony and from synchrony to stability, as expected due to the formulation of ϕ. We found no significant effect of variability in lifespan on synchrony. In addition, we found a positive effect of mean lifespan on stability as we expected (0.28). Environmentally speaking, we found a latitude gradient for both mean temperature and mean precipitation, descending towards the poles. In turn, mean temperature negatively affected stability, and annual precipitation showed a positive effect. Globally, the total effect of latitude on stability is positive but relatively small (0.15). The model using ϕ and the moving window approach (Figure 4B) explained a lower proportion of variability in stability (24%). This reduction in variance explained (7% less) was mostly because the strength of the direct effect of synchrony decreased significantly (Table S2). The direct effect of precipitation on synchrony became significant now.

Piecewise structural equations models produced contrasting results when we used different metrics, either the decomposition method (S) (Figure S10) or η (Figure S11), and whether we used synchrony calculated from the whole time series or using any of the detrend methods (Panel A or B within each figure, respectively). In the model using total synchrony (Stotal) from the decomposition method, we explained 27% of the variability in stability (Figure S10A). The main change from using ϕ is that the direct path from number of species to synchrony becomes positive (0.45, Table S3). But, its global effect on stability remains positive (0.20) due to the appearance of a new direct path from number of species to stability (0.29). Mean lifespan now has a similar positive effect on stability, and the effects of mean temperature and precipitation on stability remain. When using the synchrony from the residuals (Sdetrended) (Figure S10B) the main change is a reduction in the direct effect of synchrony on stability (from −0.21 to −0.13 and losing significance) and number of species on synchrony (from 0.45 to 0.37), the rest of the relationships remained at very similar sizes (Table S4).

When using non‐detrended η, the model explained 26% of the variability in stability (Figure S11A). As expected by the characteristics of η, which is insensitive to species number, the effect of number of species on synchrony was significantly reduced, but it also changed sign (Table S5), reflecting the pattern observed in Figure 3. The global effect of number of species on stability (0.22) remained positive due to a new direct positive path suggested by the d‐separation test (Figure S11A) and the paths through synchrony losing significance. The effects of mean lifespan on stability and the paths connecting mean annual temperature and precipitation with stability remained. Using detrended η, the model explained 23% of variability in stability (Figure S11B). By using the moving‐window approach, the direct path between number of species and stability remained but diminished (Table S6). The paths from mean lifespan and from the environmental variables to stability remained. Finally, the effect of CV Lifespan on synchrony was significant for the first time (−0.25) and a new negative path was found from mean lifespan to synchrony (−0.26).

## 4 Discussion

In the history of studies in ecological stability, a prominent attention has been paid to the patterns of synchrony in natural communities. While in theory a reduction in synchrony through compensatory dynamics should play a key role in the maintenance of stability, previous research found mostly synchronous dynamics at the interannual scale (Houlahan et al. 2007; Mutshinda et al. 2009; Valone and Barber 2008). Using an expanded pool of 124 communities of terrestrial vertebrates that account for mammals, birds, and reptiles (Figure 1), our study suggests that synchronous dynamics predominate across the globe for these taxa. Although these synchronous dynamics, likely driven by environmental forcing (Hansen et al. 2013; Mutshinda et al. 2011; Vasseur et al. 2014), contribute directly to reducing community stability, our analyses also indicate that species accumulation plays a central role in overcoming these direct effects. We specifically found statistical support for the expectation positing that the variability of the summed abundances or densities for the total of the community tends to be lower than the sum of each species' variability just by the accumulation of mismatches between the dynamics of different species (Loreau and de Mazancourt 2008; Thibaut and Connolly 2013; Xu et al. 2021). In other words, when there are more species, communities of vertebrates tend to be less synchronous because having more species increases the likelihood of attaining species with different ecological strategies. A potential limitation of this interpretation of the interplay between number of species, synchrony, and stability is the possible association between number of species and the taxonomic distance between them. Scientists tend to measure simultaneously species that are similar enough to be sampled using a common method, which may be more likely to respond synchronously to environmental cues (Larsen et al. 2025). This could potentially create an over‐representation of positive synchrony with low number of species, but the predominance of synchronous communities seems clear along the gradient of number of species (Figures 2 and 3).

Previous studies have explored the interplay between richness, synchrony, and stability (Dallas and Kramer 2021; Houlahan et al. 2007; Mutshinda et al. 2009; Valencia, de Bello, Galland, et al. 2020; Valone and Barber 2008). Here we found that the vast majority of the communities are synchronous (Figures 2 and 3) but their total abundance is yet stabilized by mechanisms of species accumulation (Figure S5, panels A and B) (Doak et al. 1998; Thibaut and Connolly 2013). Within this context, an interesting finding is that the typical case of compensatory dynamics that promote community stability through anti‐synchronous fluctuations in species population dynamics (Figure S5C) is very rare. Compensatory dynamics have been extensively studied in theoretical work (Gonzalez and Loreau 2009; Shoemaker et al. 2022), but our study suggests that it may be a rare phenomenon, or that when it happens, it gets obscured by other factors at the interannual scale. The other rare case, anti‐synchronous fluctuations and low compensation (Figure S5D), highlights the potential importance of dominance in the relationship between richness, synchrony, and stability. A small number of species, with one being very dominant, can make anti‐synchrony irrelevant for the stability of total abundance.

It is known that climate influences both population and community stability (Knape and de Valpine 2011; Stenseth et al. 2002), although this influence seems to go in different directions at the population and community levels (Dallas and Kramer 2021; Ma et al. 2017). Regardless of the metric evaluated, we generally found a negative direct effect of mean temperature on stability and a positive effect of annual precipitation on stability. Since latitude affects both variables negatively, this results in a surprisingly small positive indirect effect of latitude on stability (Figures 4, S10, and S11, Tables S1–S6). These findings therefore provide no support for the theoretical latitudinal gradient of stability, from higher stability around the equator to lower stability around the poles (Dallas and Kramer 2021; MacArthur 1955). A plausible explanation for this finding is in the characteristics of our dataset. The number of species in the communities we studied is somewhat arbitrary and it does not reflect varying levels of richness with latitude. If the latitude‐stability pattern depends mostly on statistical averaging and not on the stability of populations determined by environmental stability—as our results suggest—it will not be found in this dataset with these methods. In addition, the distribution of communities in our dataset is very uneven across latitude and thus environmental conditions, and a few communities close to the equator may be creating the pattern (Figure S3).

The positive (if relatively small) effect of mean lifespan on stability observed was consistent across metrics. Several factors could account for its small magnitude. First, our study was restricted to communities of terrestrial vertebrates. Considering a broader range of lifespans, for instance, from phytoplankton to long‐lived mammals, could provide a better signal. Second, differences in stability between communities with different mean lifespans could be hard to find when comparing series that cover only a few—if more than one—generation times for such long‐lived species. Unfortunately, such data is not available yet for a sufficiently large set of communities. Third, using only one abundance measure per year neglects seasonal variability, which is a huge source of temporal variability for short‐lived species of terrestrial vertebrates, such as rodents (Andreassen et al. 2021). Regarding the relation between variability in intra‐community lifespan and synchrony, it rarely explained much of the synchrony level observed, only short‐term correlations (Figure S11B), possibly for the reasons stated above for mean lifespan.

With respect to biotic impacts, there has been a long debate about the relative importance of richness and synchrony for community stability (Loreau and de Mazancourt 2013; McNaughton 1977; Valencia, de Bello, Galland, et al. 2020). We found a consistent positive effect of number of species on stability regardless of what metric was used and whether detrending methods were applied or not (Figures 4, S10, and S11). However, the relation between the number of species and synchrony is metric‐dependent. This reflects the fundamental difference between “stabilization” metrics like ϕ and “correlation” metrics like η. When using non‐detrended ϕ, we found the expected indirect positive effect of number of species on stability via a direct negative effect of number of species on synchrony and another direct negative effect of synchrony on stability. However, when using Stotal or non‐detrended η, the effect through synchrony on stability became very weak and negative, but the effect of number of species on stability was now mostly direct and not via synchrony, as expected because they are not affected so strongly by number of species (Gross et al. 2014; Loreau and de Mazancourt 2008). The positive effect of number of species on synchrony when using S or η may seem surprising (Figures S10 and S11). This can be explained for η by examining Figure 3, which shows that negative covariances become very difficult to achieve as the number of species increases, and for S by examining Equation (5), that shows how S tends to increase with more species if positive covariances dominate (as it is the case in our data). Put together, our results thus suggest that we should be careful when using multivariate statistical analyses such as the SEM conducted here to understand the relation between richness, synchrony, and stability, since our results may be dependent on the definition of synchrony we use and how we measure it. For example, ϕ can be problematic when comparing communities with different numbers of species. When we considered detrended synchrony metrics, the direct effect of synchrony on stability lost strength (ϕ detrended, Figure 4B), or was not significant (Sdetrended Figure S10B, η Figure S11). That means that species having opposite trends, or different long‐term dynamics of some species within a community, are creating a significant portion of the community synchrony we measure, or the lack of it (Figures S6–S8). These opposite trends are creating stability (or invariability) measured as the coefficient of variation of total abundance or density, but this may not necessarily be the type of synchrony we are looking for. This was already found in plants (Luo et al. 2025; Valencia, de Bello, Lepš, et al. 2020), and it could eventually induce an overemphasis on the importance of anti‐synchrony or compensatory dynamics when, for example, long‐term compositional changes may be the most important factor. Thus, our analyses suggest that controlling for trends in species populations is desirable when the focus is on short‐term synchrony. Different options to do that are available, such as simple detrending (Lepš et al. 2019) or timescale‐specific approaches (Zhao et al. 2020).

In conclusion, our compilation and analysis of a wide set of communities of terrestrial vertebrates with varying numbers of species has revealed that communities with synchronous dynamics are a prevalent ecological feature worldwide. This result indicates that the widely explored and assumed case of anti‐synchronous fluctuations stabilizing community dynamics is very rare, and limited to cases of low number of species, at least for communities of terrestrial vertebrates at the interannual scale. Our results have also confirmed a clear interplay between number of species and synchrony. That is, number of species offsets the negative direct effects that synchrony and mean temperature have on stability. This means that a key mechanism of community stabilization is species accumulation, which in turn accumulates imbalances between the population dynamics of the species that compose the community (Doak et al. 1998). Combining a causal model of direct and indirect effects (piecewise SEMs) with three metrics (ϕ, S, and η) revealed that the strength of the positive effect of number of species on stability is not unique. It can either produce a direct positive effect on community stability or an indirect (also positive) effect through negative direct effects from number of species to synchrony and from synchrony to stability. Therefore, if we are interested in the interplay between richness, synchrony, and stability, choosing a synchrony metric is not trivial, and the outcome needs to be interpreted carefully. For instance, while ϕ is good at detecting how several mechanisms, such as statistical averaging and anti‐synchronous fluctuations (negative covariances) make total community density or abundance more stable, η is particularly good at detecting synchronous or anti‐synchronous fluctuations of populations (positive or negative covariances, respectively). Meanwhile, detrending the time series reduces the effects of synchrony on stability, showing the importance of species population trends and long‐term dynamics such as composition changes for the stability of communities. The importance of the interplay between number of species and synchrony for community stability suggests clear ecological implications for terrestrial vertebrates. Scenarios in which global change produces novel environmental conditions under which more and more species are lost will likely reduce the stability of terrestrial vertebrate communities worldwide.

## Author Contributions

**Sergio Picó:** conceptualization (equal), data curation (lead), formal analysis (lead), investigation (lead), methodology (equal), validation (lead), visualization (lead), writing – original draft (lead), writing – review and editing (equal). **Pablo Almaraz:** conceptualization (equal), data curation (supporting), formal analysis (supporting), methodology (equal), software (equal), supervision (equal), writing – review and editing (equal). **Oscar Godoy:** conceptualization (equal), formal analysis (supporting), methodology (supporting), supervision (equal), writing – review and editing (equal).

## Funding

This work was supported by European Social Fund Plus, TASTE (PID2021‐127607OB‐I00), Universidad de Cádiz, UCA/R93REC/2019 and Ministerio de Economía y Competitividad.

## Conflicts of Interest

The authors declare no conflicts of interest.

## Supporting information


**Figure S1:** Summary of the communities studied. From left to right, histogram for number of species per community, histogram for study duration in years, and bar plot for number of communities per class of terrestrial vertebrates.
**Figure S2:** Results of the review of synchrony metrics used in a selection of ecology journals (see Methods). We classified metrics according to if they used some form of correlation or covariance (Cor/Cov), ϕ or a modification of it (Phi), a measure of the overlap of distributions (Overlap), parameter estimation (Modeling), wavelet analysis (Wavelet), or other approaches (Others).
**Figure S3:** Distribution of the communities studied throughout the Whittaker biomes. On the *x*‐axis, mean annual temperature in degrees Celsius. On the *y*‐axis, mean annual precipitation in cm. Created using the R package plotbiomes (Stefan & Levin, 2018).
**Figure S4:** Comparison of stability metrics. The observed values for 1/pv on the *y*‐axis and 1/cv on the *x*‐axis. Spearman's correlation is shown. Point size represents the number of species considered and color the length of the study in years following the same legend as Figures 1, 2, 3. The black line represents the 1:1 line.
**Figure S5:**. Comparison of synchrony metrics. Comparison between the observed synchrony values obtained for *η* (*y*‐axis) and ϕ (*x*‐axis). Percentages show the relative proportion of communities whose values lie in each quadrant. Each point represents the value for an observed community. Point size represents the number of species considered and color the length of the study in years following the same legend as Figures 1, 2, 3. The black line represents the 1:1 line.
**Figure S6:**. Effect of detrending ϕ on synchrony values observed. Comparison between the observed synchrony values obtained for non‐detrended ϕ (*y*‐axis) and detrended ϕ (*x*‐axis). Point size represents the number of species considered and color the length of the study in years, following the same legend as Figures 1, 2, 3. The black line represents the 1:1 line.
**Figure S7:**. Effect of detrending *η* on synchrony values observed. Comparison between the observed synchrony values obtained for non‐detrended *η* (*y*‐axis) and detrended *η* (*x*‐axis). Point size represents the number of species considered and color the length of the study in years following the same legend as Figures 1, 2, 3. The black line represents the 1:1 line.
**Figure S8:**. Difference between Stotal (total synchrony) and Sdetrended (synchrony from the regres sion residuals) from the decomposition method proposed in Lepš et al. (2019). Point size represents the number of species considered and color the length of the study in years following the same legend as Figures 1, 2, 3. The black line represents the 1:1 line.
**Figure S9:** A priori causal model. Causal model used as a starting point for every piecewise structural equation model.
**Figure S10:** Piecewise structural equations models using synchrony calculated with the decomposition method in Lepš et al. (2019). (A) Model using total synchrony (Stotal). (B) Model using synchrony from the residuals (Sdetrended). Solid lines represent positive effects while dashed lines represent negative effects. Black lines represent significant effects according to the tests of d‐separation, gray lines represent non‐significant effects. The width of each arrow is proportional to the corresponding standardized path coefficient.
**Figure S11:**. Piecewise structural equations models using *η* as synchrony metric. (A) Model using non‐detrended *η*. (B) Model using detrended *η*. Solid lines represent positive effects while dashed lines represent negative effects. Black lines represent significant effects according to the tests of d separation, gray lines represent non‐significant effects. The width of each arrow is proportional to the corresponding standardized path coefficient.
**Table S1:**. Standardized path coefficients from the model using non‐detrended ϕ (Figure 4A). Fisher's *C* = 22.927, *p* = 0.524, df = 24.
**Table S2:** Standardized path coefficients from the model using detrended ϕ (Figure 4B). Fisher's *C* = 17.106, *p* = 0.844, df = 24.
**Table S3:** Standardized path coefficients from the model using total synchrony (Stotal), following the decomposition method in Lepš et al.(2019). Fisher's *C* = 15.110, *p* = 0.857, df = 22.
**Table S4:**. Standardized path coefficients from the model using synchrony of residuals after detrending by linear regression (Sdetrended), following the method in Lepš et al. (2019). Fisher's *C* = 17.512, *p* = 0.735, df = 22.
**Table S5:** Standardized path coefficients from the model using non‐detrended *η* (Figure S11A). Fisher's *C* = 16.954, *p* = 0.766, df = 22.
**Table S6:**. Standardized path coefficients from the model using detrended *η* (Figure S11B). Fisher's *C* = 12.161, *p* = 0.910, df = 20.

## Data Availability

Data and code needed to reproduce the results of this study are stored in a public repository: https://doi.org/10.6084/m9.figshare.30455942.
