# Supplementary figures and images for: Species Accumulation Stabilizes the Synchronous Responses to the Environment of Vertebrate Communities Worldwide
Source: Ecol Evol. 2026 Jun 21;16(6):e73723. doi: 10.1002/ece3.73723 (PMC13283458; doi:10.1002/ece3.73723)

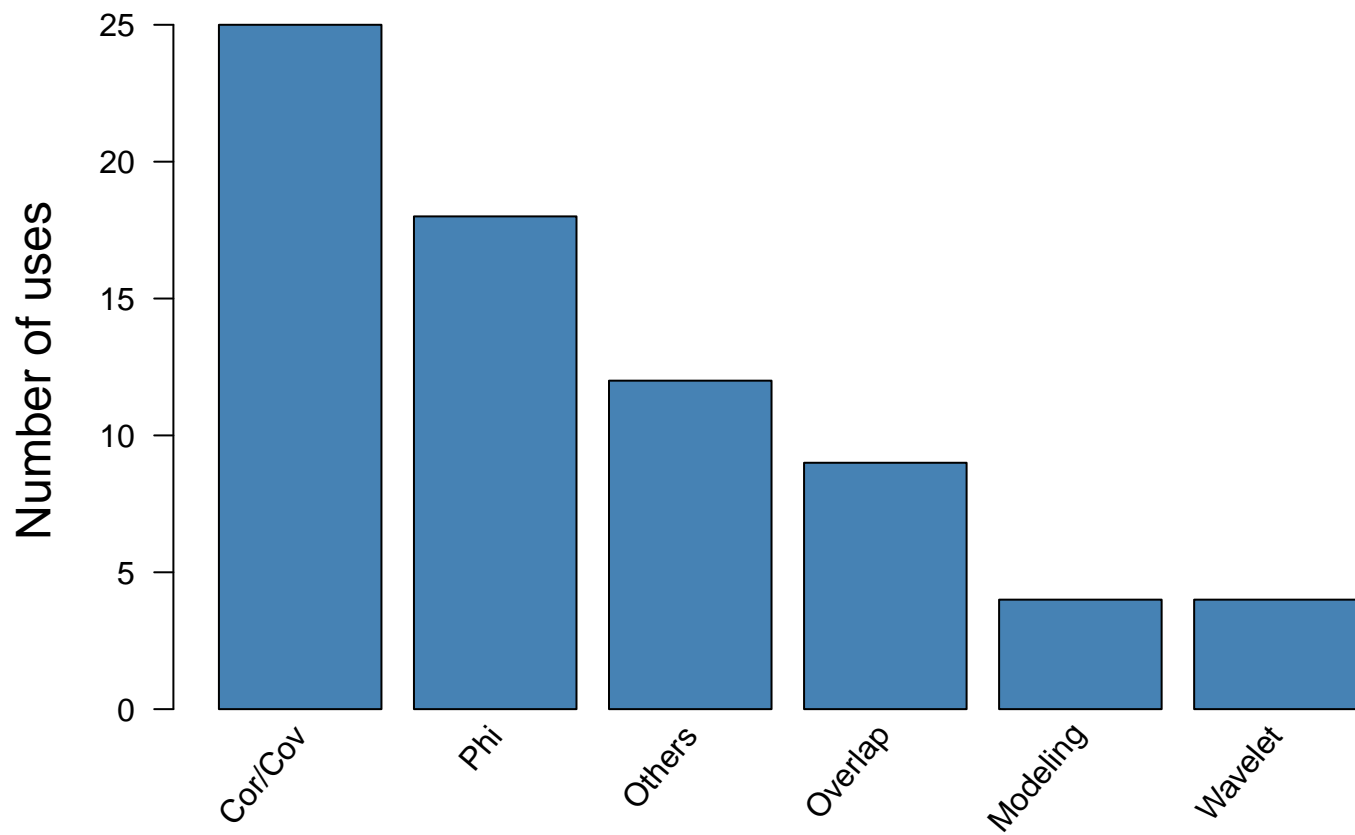

Supplement: Supplementary file 1 — Figure S1: Summary of the communities studied. From left to right, histogram for number of species per community, histogram for study duration in years, and bar plot for number of communities per class of terrestrial vertebrates. Figure S2: Results of the review of synchrony metrics used in a selection of ecology journals (see Methods). We classified metrics according to if they used some form of correlation or covariance (Cor/Cov), ϕ or a modification of it (Phi), a measure of the overlap of distributions (Overlap), parameter estimation (Modeling), wavelet analysis (Wavelet), or other approaches (Others). Figure S3: Distribution of the communities studied throughout the Whittaker biomes. On the x‐axis, mean annual temperature in degrees Celsius. On the y‐axis, mean annual precipitation in cm. Created using the R package plotbiomes (Stefan & Levin, 2018). Figure S4: Comparison of stability metrics. The observed values for 1/pv on the y‐axis and 1/cv on the x‐axis. Spearman's correlation is shown. Point size represents the number of species considered and color the length of the study in years following the same legend as Figures 1, 2, 3. The black line represents the 1:1 line. Figure S5:. Comparison of synchrony metrics. Comparison between the observed synchrony values obtained for η (y‐axis) and ϕ (x‐axis). Percentages show the relative proportion of communities whose values lie in each quadrant. Each point represents the value for an observed community. Point size represents the number of species considered and color the length of the study in years following the same legend as Figures 1, 2, 3. The black line represents the 1:1 line. Figure S6:. Effect of detrending ϕ on synchrony values observed. Comparison between the observed synchrony values obtained for non‐detrended ϕ (y‐axis) and detrended ϕ (x‐axis). Point size represents the number of species considered and color the length of the study in years, following the same legend as Figures 1, 2, 3. The black lin [file ECE3-16-e73723-s001.zip › Figure S2.pdf]

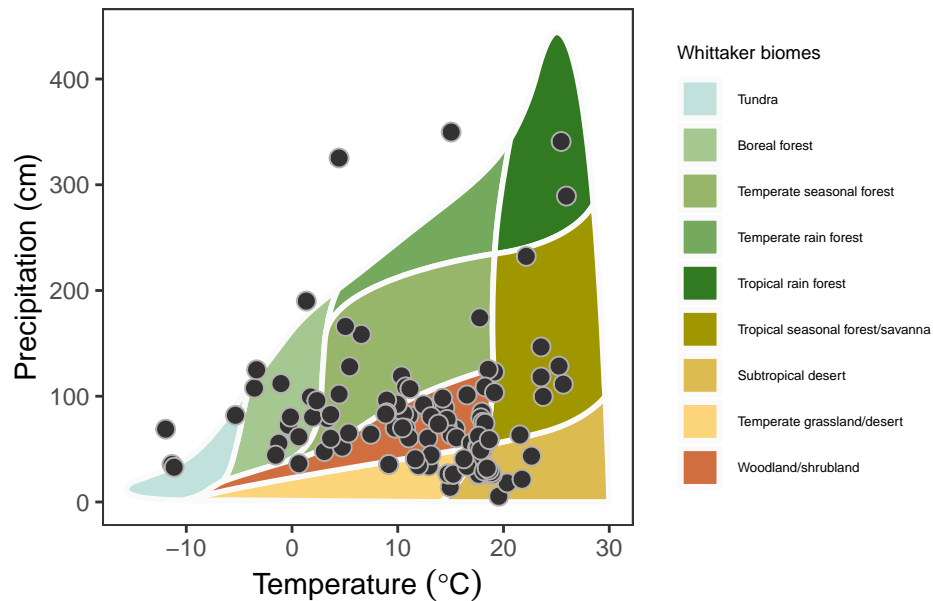

Supplement: Supplementary file 1 — Figure S1: Summary of the communities studied. From left to right, histogram for number of species per community, histogram for study duration in years, and bar plot for number of communities per class of terrestrial vertebrates. Figure S2: Results of the review of synchrony metrics used in a selection of ecology journals (see Methods). We classified metrics according to if they used some form of correlation or covariance (Cor/Cov), ϕ or a modification of it (Phi), a measure of the overlap of distributions (Overlap), parameter estimation (Modeling), wavelet analysis (Wavelet), or other approaches (Others). Figure S3: Distribution of the communities studied throughout the Whittaker biomes. On the x‐axis, mean annual temperature in degrees Celsius. On the y‐axis, mean annual precipitation in cm. Created using the R package plotbiomes (Stefan & Levin, 2018). Figure S4: Comparison of stability metrics. The observed values for 1/pv on the y‐axis and 1/cv on the x‐axis. Spearman's correlation is shown. Point size represents the number of species considered and color the length of the study in years following the same legend as Figures 1, 2, 3. The black line represents the 1:1 line. Figure S5:. Comparison of synchrony metrics. Comparison between the observed synchrony values obtained for η (y‐axis) and ϕ (x‐axis). Percentages show the relative proportion of communities whose values lie in each quadrant. Each point represents the value for an observed community. Point size represents the number of species considered and color the length of the study in years following the same legend as Figures 1, 2, 3. The black line represents the 1:1 line. Figure S6:. Effect of detrending ϕ on synchrony values observed. Comparison between the observed synchrony values obtained for non‐detrended ϕ (y‐axis) and detrended ϕ (x‐axis). Point size represents the number of species considered and color the length of the study in years, following the same legend as Figures 1, 2, 3. The black lin [file ECE3-16-e73723-s001.zip › Figure S3.pdf]

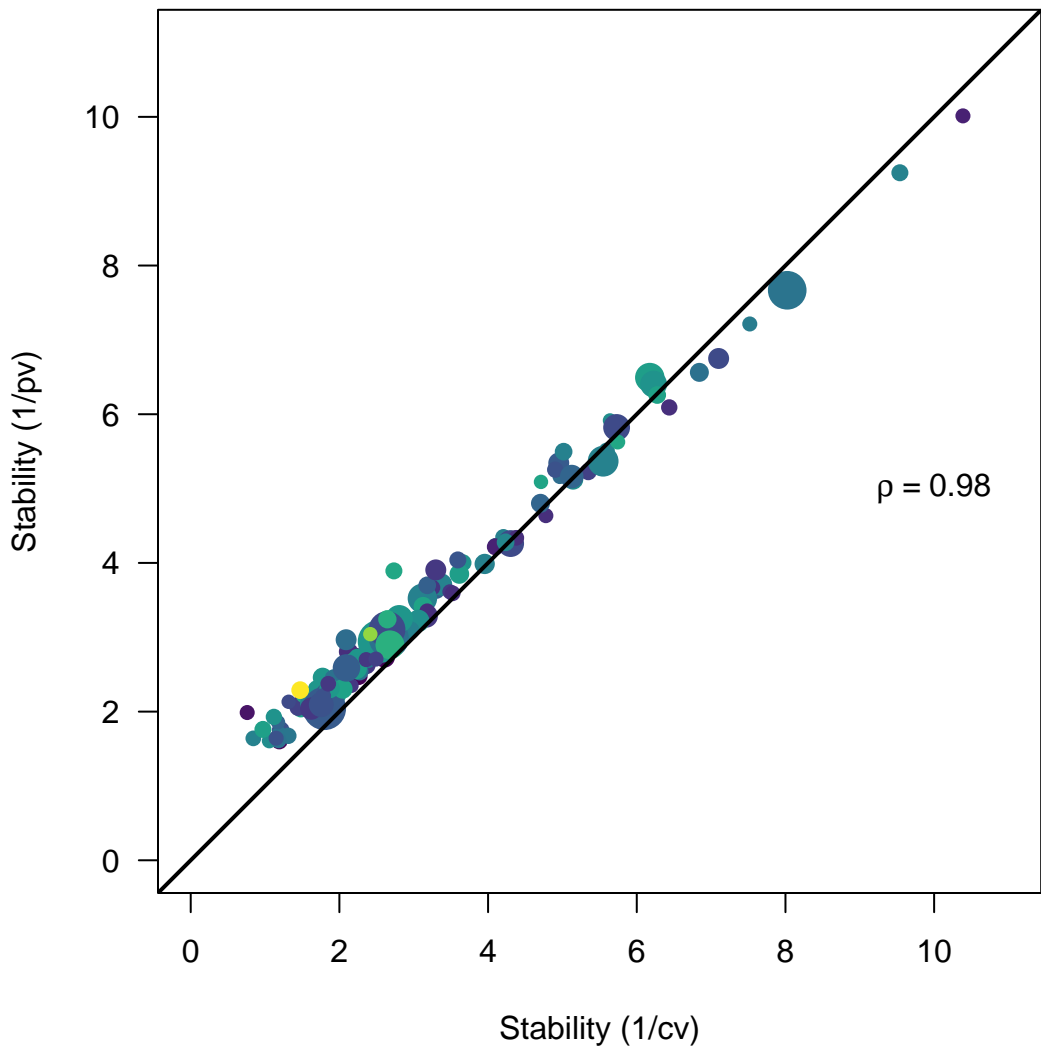

Supplement: Supplementary file 1 — Figure S1: Summary of the communities studied. From left to right, histogram for number of species per community, histogram for study duration in years, and bar plot for number of communities per class of terrestrial vertebrates. Figure S2: Results of the review of synchrony metrics used in a selection of ecology journals (see Methods). We classified metrics according to if they used some form of correlation or covariance (Cor/Cov), ϕ or a modification of it (Phi), a measure of the overlap of distributions (Overlap), parameter estimation (Modeling), wavelet analysis (Wavelet), or other approaches (Others). Figure S3: Distribution of the communities studied throughout the Whittaker biomes. On the x‐axis, mean annual temperature in degrees Celsius. On the y‐axis, mean annual precipitation in cm. Created using the R package plotbiomes (Stefan & Levin, 2018). Figure S4: Comparison of stability metrics. The observed values for 1/pv on the y‐axis and 1/cv on the x‐axis. Spearman's correlation is shown. Point size represents the number of species considered and color the length of the study in years following the same legend as Figures 1, 2, 3. The black line represents the 1:1 line. Figure S5:. Comparison of synchrony metrics. Comparison between the observed synchrony values obtained for η (y‐axis) and ϕ (x‐axis). Percentages show the relative proportion of communities whose values lie in each quadrant. Each point represents the value for an observed community. Point size represents the number of species considered and color the length of the study in years following the same legend as Figures 1, 2, 3. The black line represents the 1:1 line. Figure S6:. Effect of detrending ϕ on synchrony values observed. Comparison between the observed synchrony values obtained for non‐detrended ϕ (y‐axis) and detrended ϕ (x‐axis). Point size represents the number of species considered and color the length of the study in years, following the same legend as Figures 1, 2, 3. The black lin [file ECE3-16-e73723-s001.zip › Figure S4.pdf]

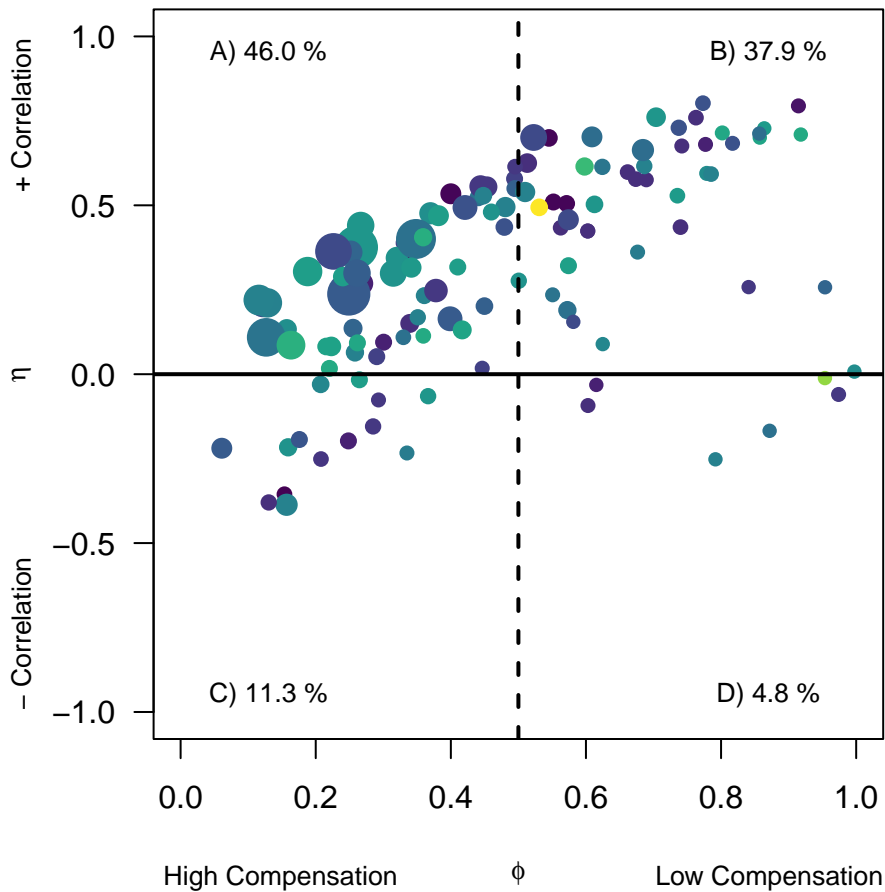

Supplement: Supplementary file 1 — Figure S1: Summary of the communities studied. From left to right, histogram for number of species per community, histogram for study duration in years, and bar plot for number of communities per class of terrestrial vertebrates. Figure S2: Results of the review of synchrony metrics used in a selection of ecology journals (see Methods). We classified metrics according to if they used some form of correlation or covariance (Cor/Cov), ϕ or a modification of it (Phi), a measure of the overlap of distributions (Overlap), parameter estimation (Modeling), wavelet analysis (Wavelet), or other approaches (Others). Figure S3: Distribution of the communities studied throughout the Whittaker biomes. On the x‐axis, mean annual temperature in degrees Celsius. On the y‐axis, mean annual precipitation in cm. Created using the R package plotbiomes (Stefan & Levin, 2018). Figure S4: Comparison of stability metrics. The observed values for 1/pv on the y‐axis and 1/cv on the x‐axis. Spearman's correlation is shown. Point size represents the number of species considered and color the length of the study in years following the same legend as Figures 1, 2, 3. The black line represents the 1:1 line. Figure S5:. Comparison of synchrony metrics. Comparison between the observed synchrony values obtained for η (y‐axis) and ϕ (x‐axis). Percentages show the relative proportion of communities whose values lie in each quadrant. Each point represents the value for an observed community. Point size represents the number of species considered and color the length of the study in years following the same legend as Figures 1, 2, 3. The black line represents the 1:1 line. Figure S6:. Effect of detrending ϕ on synchrony values observed. Comparison between the observed synchrony values obtained for non‐detrended ϕ (y‐axis) and detrended ϕ (x‐axis). Point size represents the number of species considered and color the length of the study in years, following the same legend as Figures 1, 2, 3. The black lin [file ECE3-16-e73723-s001.zip › Figure S5.pdf]

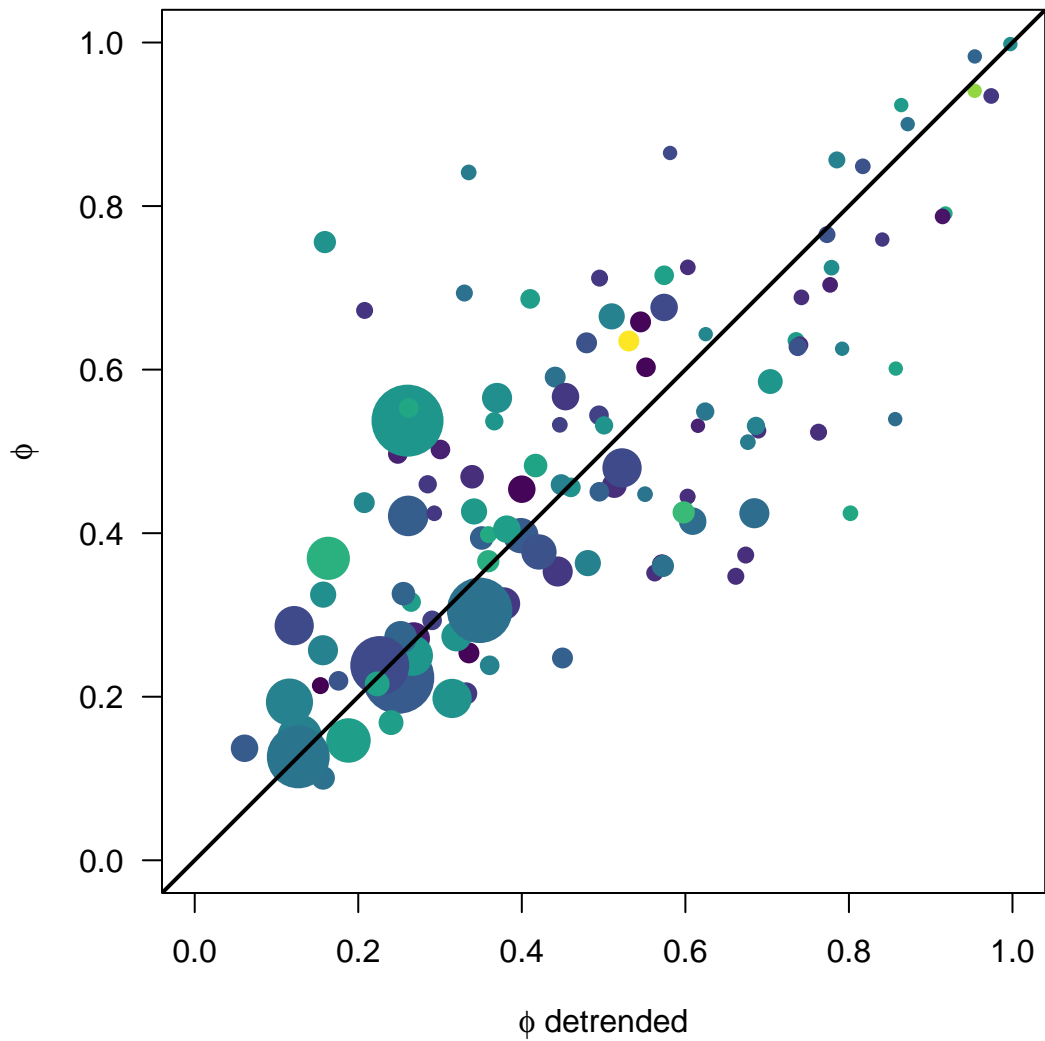

Supplement: Supplementary file 1 — Figure S1: Summary of the communities studied. From left to right, histogram for number of species per community, histogram for study duration in years, and bar plot for number of communities per class of terrestrial vertebrates. Figure S2: Results of the review of synchrony metrics used in a selection of ecology journals (see Methods). We classified metrics according to if they used some form of correlation or covariance (Cor/Cov), ϕ or a modification of it (Phi), a measure of the overlap of distributions (Overlap), parameter estimation (Modeling), wavelet analysis (Wavelet), or other approaches (Others). Figure S3: Distribution of the communities studied throughout the Whittaker biomes. On the x‐axis, mean annual temperature in degrees Celsius. On the y‐axis, mean annual precipitation in cm. Created using the R package plotbiomes (Stefan & Levin, 2018). Figure S4: Comparison of stability metrics. The observed values for 1/pv on the y‐axis and 1/cv on the x‐axis. Spearman's correlation is shown. Point size represents the number of species considered and color the length of the study in years following the same legend as Figures 1, 2, 3. The black line represents the 1:1 line. Figure S5:. Comparison of synchrony metrics. Comparison between the observed synchrony values obtained for η (y‐axis) and ϕ (x‐axis). Percentages show the relative proportion of communities whose values lie in each quadrant. Each point represents the value for an observed community. Point size represents the number of species considered and color the length of the study in years following the same legend as Figures 1, 2, 3. The black line represents the 1:1 line. Figure S6:. Effect of detrending ϕ on synchrony values observed. Comparison between the observed synchrony values obtained for non‐detrended ϕ (y‐axis) and detrended ϕ (x‐axis). Point size represents the number of species considered and color the length of the study in years, following the same legend as Figures 1, 2, 3. The black lin [file ECE3-16-e73723-s001.zip › Figure S6.pdf]

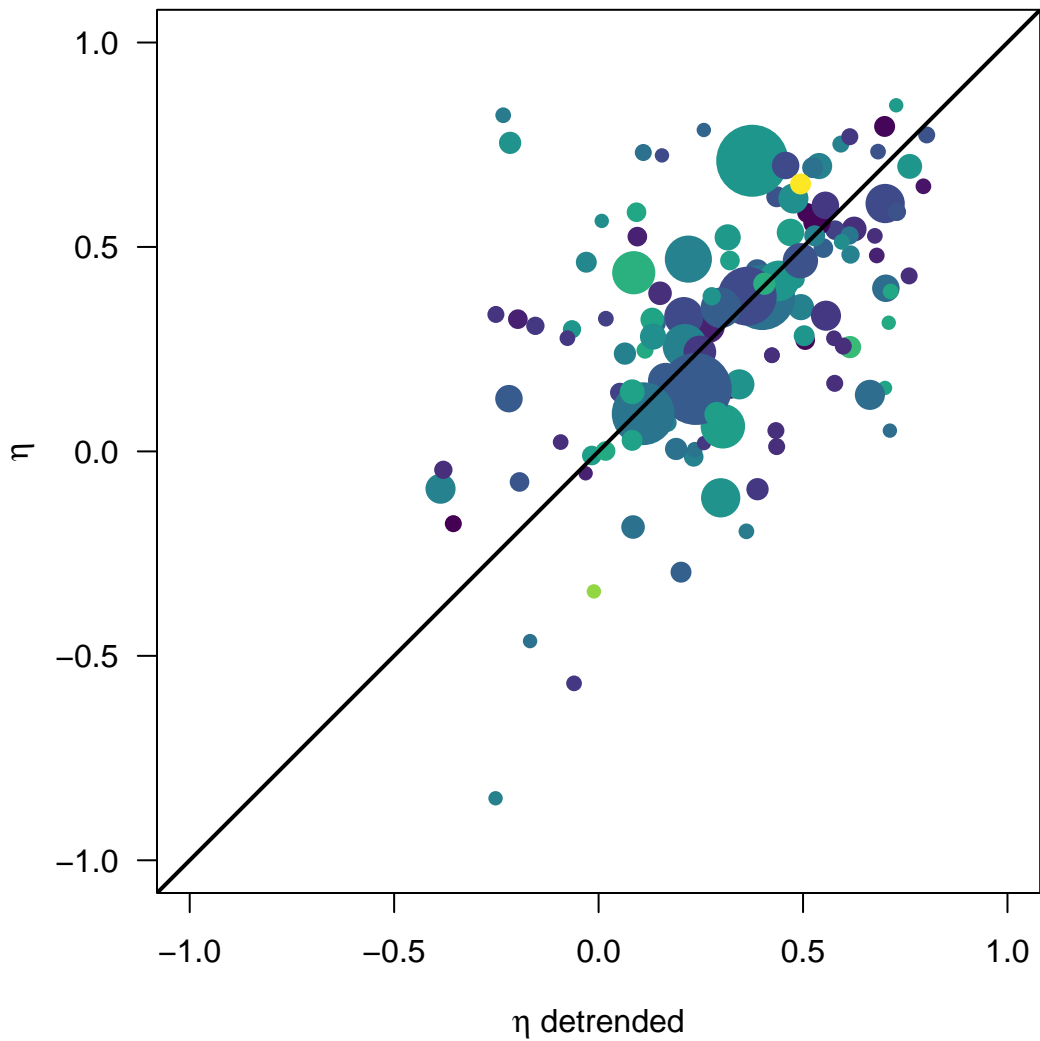

Supplement: Supplementary file 1 — Figure S1: Summary of the communities studied. From left to right, histogram for number of species per community, histogram for study duration in years, and bar plot for number of communities per class of terrestrial vertebrates. Figure S2: Results of the review of synchrony metrics used in a selection of ecology journals (see Methods). We classified metrics according to if they used some form of correlation or covariance (Cor/Cov), ϕ or a modification of it (Phi), a measure of the overlap of distributions (Overlap), parameter estimation (Modeling), wavelet analysis (Wavelet), or other approaches (Others). Figure S3: Distribution of the communities studied throughout the Whittaker biomes. On the x‐axis, mean annual temperature in degrees Celsius. On the y‐axis, mean annual precipitation in cm. Created using the R package plotbiomes (Stefan & Levin, 2018). Figure S4: Comparison of stability metrics. The observed values for 1/pv on the y‐axis and 1/cv on the x‐axis. Spearman's correlation is shown. Point size represents the number of species considered and color the length of the study in years following the same legend as Figures 1, 2, 3. The black line represents the 1:1 line. Figure S5:. Comparison of synchrony metrics. Comparison between the observed synchrony values obtained for η (y‐axis) and ϕ (x‐axis). Percentages show the relative proportion of communities whose values lie in each quadrant. Each point represents the value for an observed community. Point size represents the number of species considered and color the length of the study in years following the same legend as Figures 1, 2, 3. The black line represents the 1:1 line. Figure S6:. Effect of detrending ϕ on synchrony values observed. Comparison between the observed synchrony values obtained for non‐detrended ϕ (y‐axis) and detrended ϕ (x‐axis). Point size represents the number of species considered and color the length of the study in years, following the same legend as Figures 1, 2, 3. The black lin [file ECE3-16-e73723-s001.zip › Figure S7.pdf]

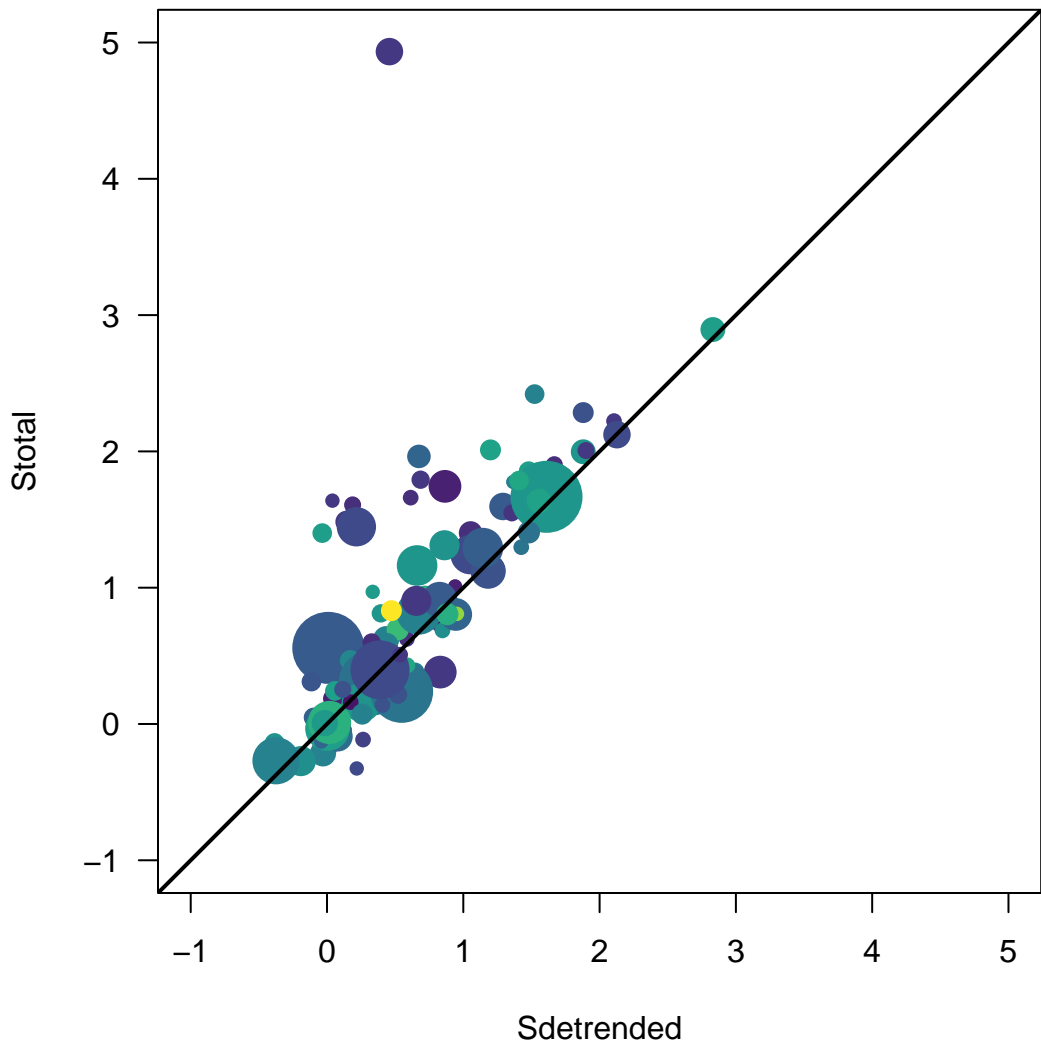

Supplement: Supplementary file 1 — Figure S1: Summary of the communities studied. From left to right, histogram for number of species per community, histogram for study duration in years, and bar plot for number of communities per class of terrestrial vertebrates. Figure S2: Results of the review of synchrony metrics used in a selection of ecology journals (see Methods). We classified metrics according to if they used some form of correlation or covariance (Cor/Cov), ϕ or a modification of it (Phi), a measure of the overlap of distributions (Overlap), parameter estimation (Modeling), wavelet analysis (Wavelet), or other approaches (Others). Figure S3: Distribution of the communities studied throughout the Whittaker biomes. On the x‐axis, mean annual temperature in degrees Celsius. On the y‐axis, mean annual precipitation in cm. Created using the R package plotbiomes (Stefan & Levin, 2018). Figure S4: Comparison of stability metrics. The observed values for 1/pv on the y‐axis and 1/cv on the x‐axis. Spearman's correlation is shown. Point size represents the number of species considered and color the length of the study in years following the same legend as Figures 1, 2, 3. The black line represents the 1:1 line. Figure S5:. Comparison of synchrony metrics. Comparison between the observed synchrony values obtained for η (y‐axis) and ϕ (x‐axis). Percentages show the relative proportion of communities whose values lie in each quadrant. Each point represents the value for an observed community. Point size represents the number of species considered and color the length of the study in years following the same legend as Figures 1, 2, 3. The black line represents the 1:1 line. Figure S6:. Effect of detrending ϕ on synchrony values observed. Comparison between the observed synchrony values obtained for non‐detrended ϕ (y‐axis) and detrended ϕ (x‐axis). Point size represents the number of species considered and color the length of the study in years, following the same legend as Figures 1, 2, 3. The black lin [file ECE3-16-e73723-s001.zip › Figure S8.pdf]

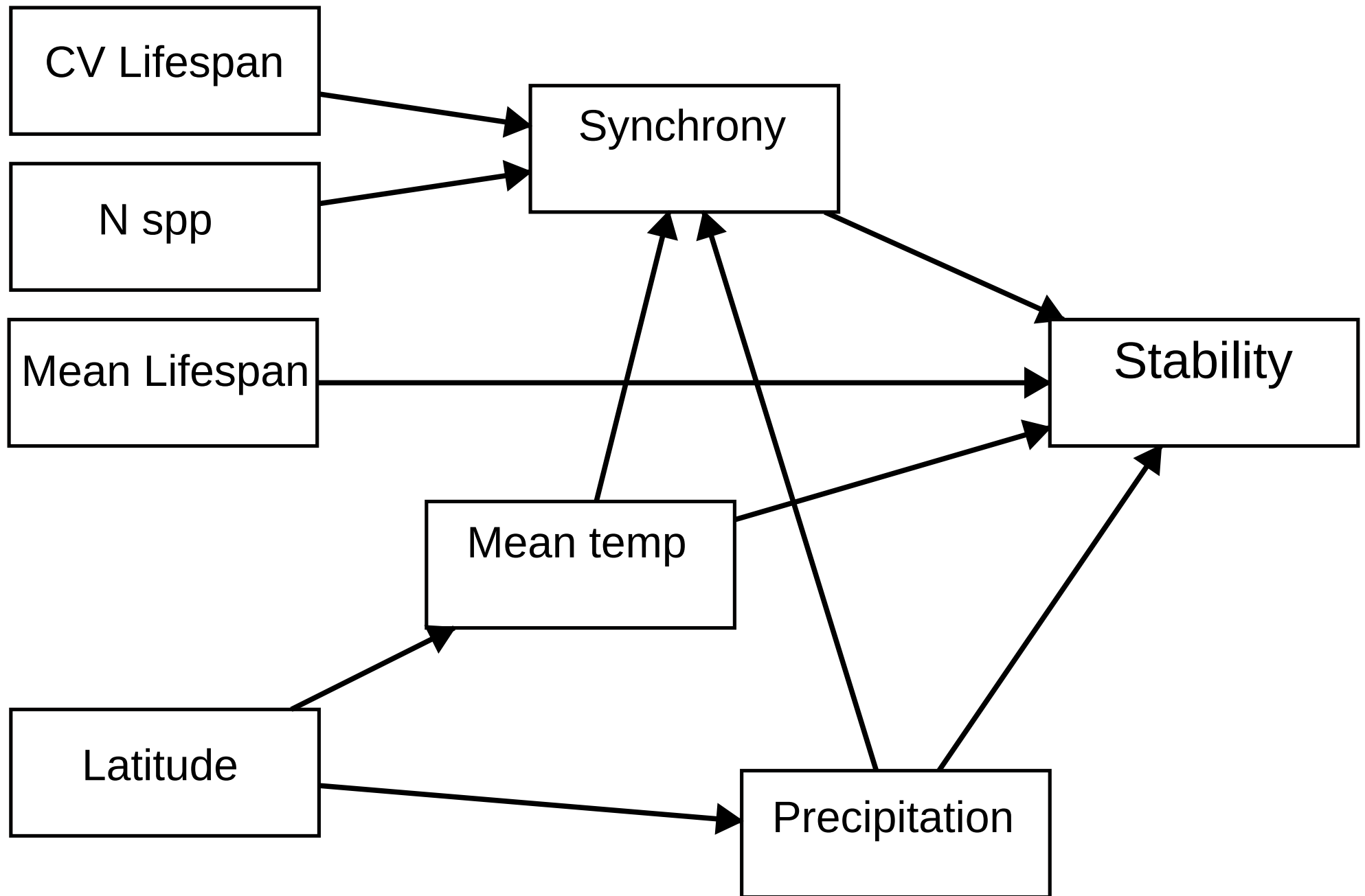

Supplement: Supplementary file 1 — Figure S1: Summary of the communities studied. From left to right, histogram for number of species per community, histogram for study duration in years, and bar plot for number of communities per class of terrestrial vertebrates. Figure S2: Results of the review of synchrony metrics used in a selection of ecology journals (see Methods). We classified metrics according to if they used some form of correlation or covariance (Cor/Cov), ϕ or a modification of it (Phi), a measure of the overlap of distributions (Overlap), parameter estimation (Modeling), wavelet analysis (Wavelet), or other approaches (Others). Figure S3: Distribution of the communities studied throughout the Whittaker biomes. On the x‐axis, mean annual temperature in degrees Celsius. On the y‐axis, mean annual precipitation in cm. Created using the R package plotbiomes (Stefan & Levin, 2018). Figure S4: Comparison of stability metrics. The observed values for 1/pv on the y‐axis and 1/cv on the x‐axis. Spearman's correlation is shown. Point size represents the number of species considered and color the length of the study in years following the same legend as Figures 1, 2, 3. The black line represents the 1:1 line. Figure S5:. Comparison of synchrony metrics. Comparison between the observed synchrony values obtained for η (y‐axis) and ϕ (x‐axis). Percentages show the relative proportion of communities whose values lie in each quadrant. Each point represents the value for an observed community. Point size represents the number of species considered and color the length of the study in years following the same legend as Figures 1, 2, 3. The black line represents the 1:1 line. Figure S6:. Effect of detrending ϕ on synchrony values observed. Comparison between the observed synchrony values obtained for non‐detrended ϕ (y‐axis) and detrended ϕ (x‐axis). Point size represents the number of species considered and color the length of the study in years, following the same legend as Figures 1, 2, 3. The black lin [file ECE3-16-e73723-s001.zip › Figure S9.pdf]

A)

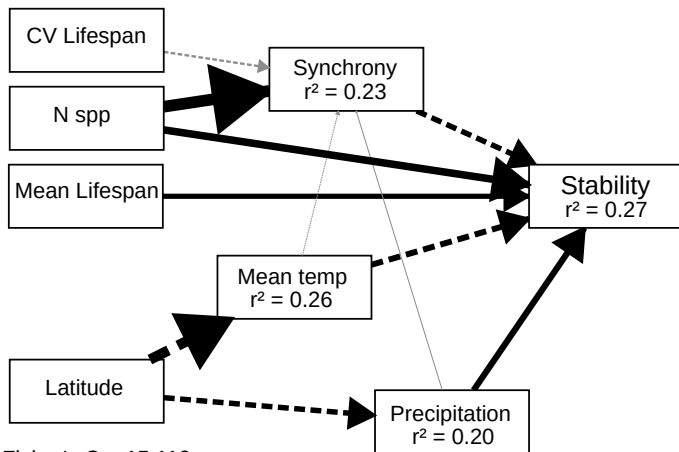

Fisher's C = 15.110  
p-value = 0.857

B)

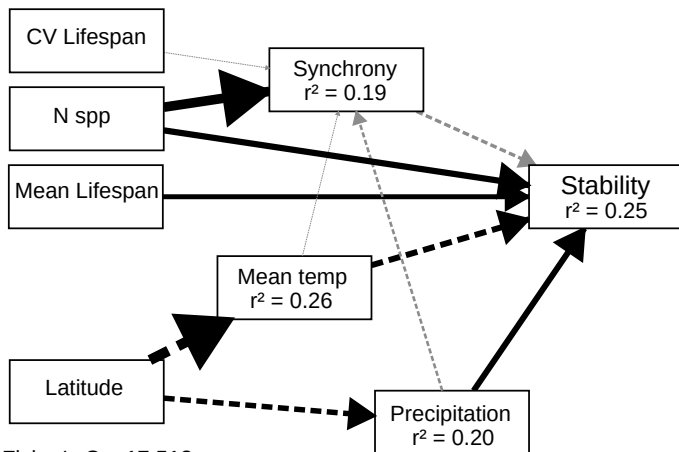

Fisher's C = 17.512  
p-value = 0.735

Supplement: Supplementary file 1 — Figure S1: Summary of the communities studied. From left to right, histogram for number of species per community, histogram for study duration in years, and bar plot for number of communities per class of terrestrial vertebrates. Figure S2: Results of the review of synchrony metrics used in a selection of ecology journals (see Methods). We classified metrics according to if they used some form of correlation or covariance (Cor/Cov), ϕ or a modification of it (Phi), a measure of the overlap of distributions (Overlap), parameter estimation (Modeling), wavelet analysis (Wavelet), or other approaches (Others). Figure S3: Distribution of the communities studied throughout the Whittaker biomes. On the x‐axis, mean annual temperature in degrees Celsius. On the y‐axis, mean annual precipitation in cm. Created using the R package plotbiomes (Stefan & Levin, 2018). Figure S4: Comparison of stability metrics. The observed values for 1/pv on the y‐axis and 1/cv on the x‐axis. Spearman's correlation is shown. Point size represents the number of species considered and color the length of the study in years following the same legend as Figures 1, 2, 3. The black line represents the 1:1 line. Figure S5:. Comparison of synchrony metrics. Comparison between the observed synchrony values obtained for η (y‐axis) and ϕ (x‐axis). Percentages show the relative proportion of communities whose values lie in each quadrant. Each point represents the value for an observed community. Point size represents the number of species considered and color the length of the study in years following the same legend as Figures 1, 2, 3. The black line represents the 1:1 line. Figure S6:. Effect of detrending ϕ on synchrony values observed. Comparison between the observed synchrony values obtained for non‐detrended ϕ (y‐axis) and detrended ϕ (x‐axis). Point size represents the number of species considered and color the length of the study in years, following the same legend as Figures 1, 2, 3. The black lin [file ECE3-16-e73723-s001.zip › Figure S10.pdf]

A)

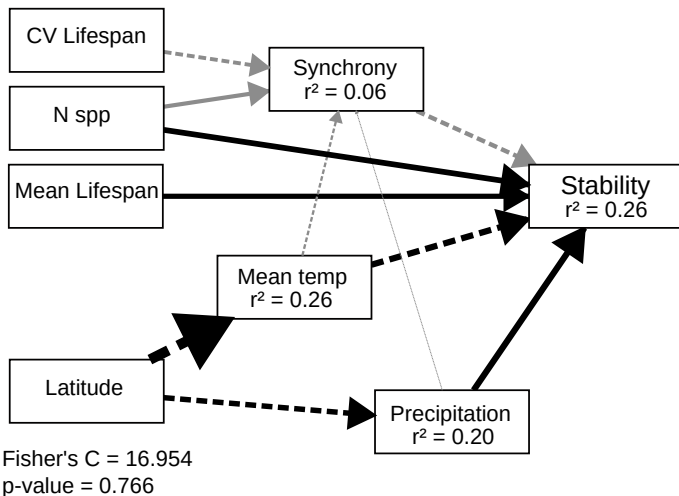

B)

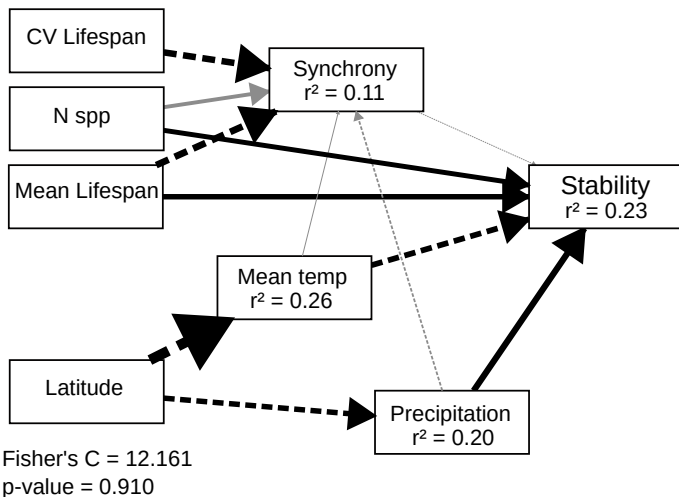

Supplement: Supplementary file 1 — Figure S1: Summary of the communities studied. From left to right, histogram for number of species per community, histogram for study duration in years, and bar plot for number of communities per class of terrestrial vertebrates. Figure S2: Results of the review of synchrony metrics used in a selection of ecology journals (see Methods). We classified metrics according to if they used some form of correlation or covariance (Cor/Cov), ϕ or a modification of it (Phi), a measure of the overlap of distributions (Overlap), parameter estimation (Modeling), wavelet analysis (Wavelet), or other approaches (Others). Figure S3: Distribution of the communities studied throughout the Whittaker biomes. On the x‐axis, mean annual temperature in degrees Celsius. On the y‐axis, mean annual precipitation in cm. Created using the R package plotbiomes (Stefan & Levin, 2018). Figure S4: Comparison of stability metrics. The observed values for 1/pv on the y‐axis and 1/cv on the x‐axis. Spearman's correlation is shown. Point size represents the number of species considered and color the length of the study in years following the same legend as Figures 1, 2, 3. The black line represents the 1:1 line. Figure S5:. Comparison of synchrony metrics. Comparison between the observed synchrony values obtained for η (y‐axis) and ϕ (x‐axis). Percentages show the relative proportion of communities whose values lie in each quadrant. Each point represents the value for an observed community. Point size represents the number of species considered and color the length of the study in years following the same legend as Figures 1, 2, 3. The black line represents the 1:1 line. Figure S6:. Effect of detrending ϕ on synchrony values observed. Comparison between the observed synchrony values obtained for non‐detrended ϕ (y‐axis) and detrended ϕ (x‐axis). Point size represents the number of species considered and color the length of the study in years, following the same legend as Figures 1, 2, 3. The black lin [file ECE3-16-e73723-s001.zip › Figure S11.pdf]

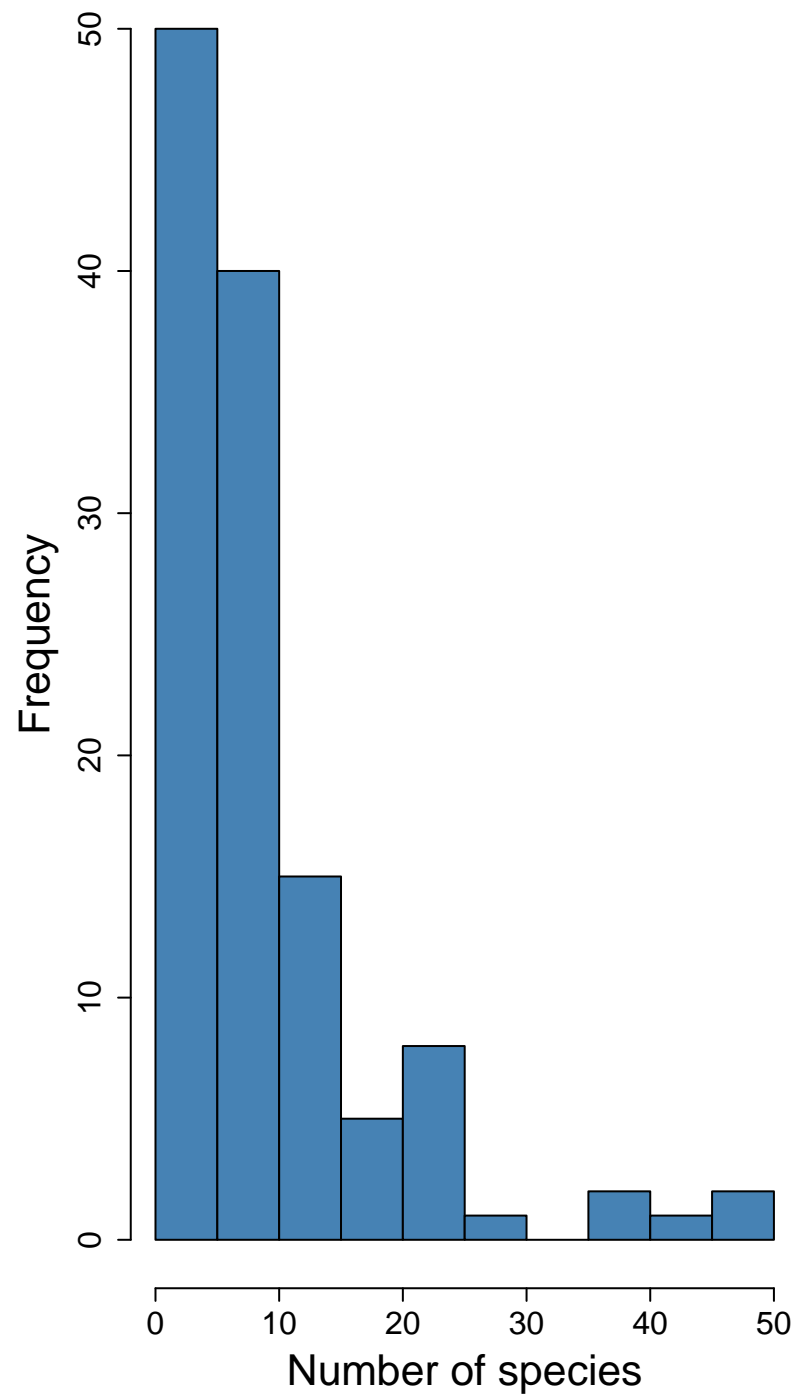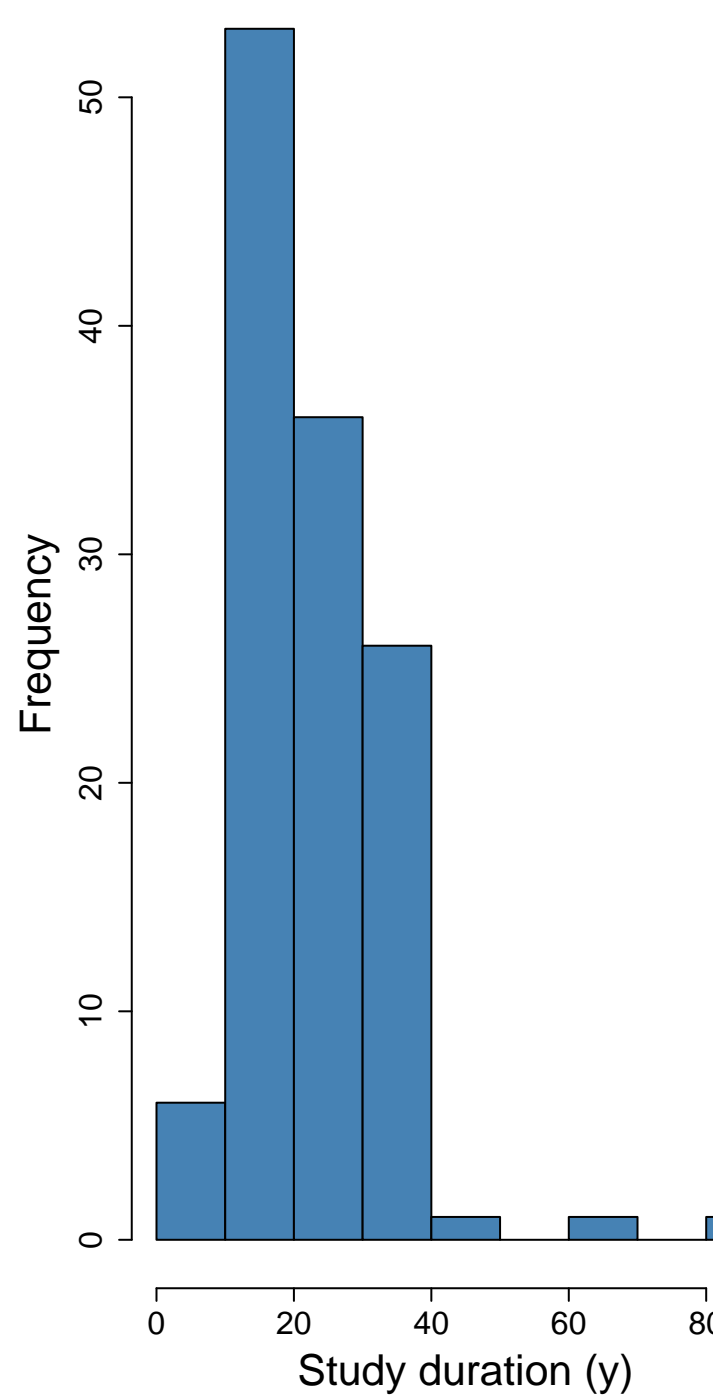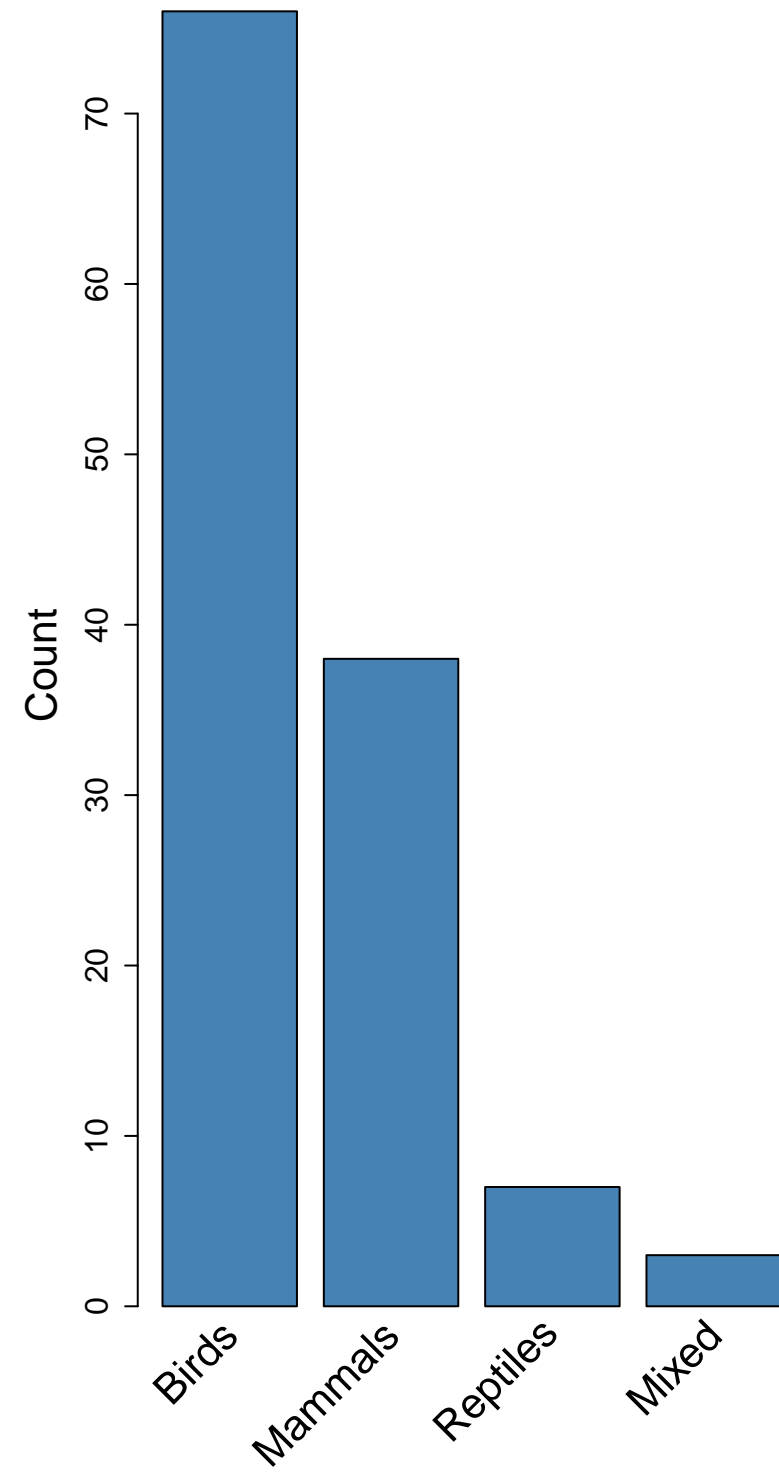

Supplement: Supplementary file 1 — Figure S1: Summary of the communities studied. From left to right, histogram for number of species per community, histogram for study duration in years, and bar plot for number of communities per class of terrestrial vertebrates. Figure S2: Results of the review of synchrony metrics used in a selection of ecology journals (see Methods). We classified metrics according to if they used some form of correlation or covariance (Cor/Cov), ϕ or a modification of it (Phi), a measure of the overlap of distributions (Overlap), parameter estimation (Modeling), wavelet analysis (Wavelet), or other approaches (Others). Figure S3: Distribution of the communities studied throughout the Whittaker biomes. On the x‐axis, mean annual temperature in degrees Celsius. On the y‐axis, mean annual precipitation in cm. Created using the R package plotbiomes (Stefan & Levin, 2018). Figure S4: Comparison of stability metrics. The observed values for 1/pv on the y‐axis and 1/cv on the x‐axis. Spearman's correlation is shown. Point size represents the number of species considered and color the length of the study in years following the same legend as Figures 1, 2, 3. The black line represents the 1:1 line. Figure S5:. Comparison of synchrony metrics. Comparison between the observed synchrony values obtained for η (y‐axis) and ϕ (x‐axis). Percentages show the relative proportion of communities whose values lie in each quadrant. Each point represents the value for an observed community. Point size represents the number of species considered and color the length of the study in years following the same legend as Figures 1, 2, 3. The black line represents the 1:1 line. Figure S6:. Effect of detrending ϕ on synchrony values observed. Comparison between the observed synchrony values obtained for non‐detrended ϕ (y‐axis) and detrended ϕ (x‐axis). Point size represents the number of species considered and color the length of the study in years, following the same legend as Figures 1, 2, 3. The black lin [file ECE3-16-e73723-s001.zip › Figure S1.pdf]
